# Development of chemical categories for per- and polyfluoroalkyl substances (PFAS) and the proof-of-concept approach to the identification of potential candidates for tiered toxicological testing and human health assessment

**DOI:** 10.1016/j.comtox.2024.100327

**Published:** 2024-09-01

**Authors:** G. Patlewicz, R.S. Judson, A.J. Williams, T. Butler, S. Barone, K.E. Carstens, J. Cowden, J.L. Dawson, S.J. Degitz, K. Fay, T.R. Henry, A. Lowit, S. Padilla, K. Paul Friedman, M.B. Phillips, D. Turk, J.F. Wambaugh, B.A. Wetmore, R.S. Thomas

**Affiliations:** a Center for Computational Toxicology & Exposure (CCTE), U.S. Environmental Protection Agency, Research Triangle Park, Durham, NC 27709, USA; b Office of Chemical Safety and Pollution Prevention (OSCPP), US Environmental Protection Agency, DC, USA

**Keywords:** Per- and polyfluoroalkyl substances (PFAS), Chemical categories, Read-across, New Approach Methods (NAMs), Data collection, Toxic Substances Control Act (TSCA)

## Abstract

Per- and Polyfluoroalkyl substances (PFAS) are a class of manufactured chemicals that are in widespread use and many present concerns for persistence, bioaccumulation and toxicity. Whilst a handful of PFAS have been characterized for their hazard profiles, the vast majority have not been extensively studied. Herein, a chemical category approach was developed and applied to PFAS that could be readily characterized by a chemical structure. The PFAS definition as described in the Toxic Substances Control Act (TSCA) section 8(a)(7) rule was applied to the Distributed Structure-Searchable Toxicity (DSSTox) database to retrieve an initial list of 13,054 PFAS. Plausible degradation products from the 563 PFAS on the non-confidential TSCA Inventory were simulated using the Catalogic expert system, and the unique predicted PFAS degradants (2484) that conformed to the same PFAS definition were added to the list resulting in a set of 15,538 PFAS. Each PFAS was then assigned into a primary category using Organisation for Economic Co-operation and Development (OECD) structure-based classifications. The primary categories were subdivided into secondary categories based on a chain length threshold (>=7 vs < 7). Secondary categories were subcategorized using chemical fingerprints to achieve a balance between total number of structural categories vs. level of structural similarity within a category based on the Jaccard index. A set of 128 terminal structural categories were derived from which a subset of representative candidates could be proposed for potential data collection, considering the sparsity of relevant toxicity data within each category, presence on environmental monitoring lists, and the ability to identify plausible manufacturers/importers. Refinements to the approach taking into consideration ways in which the categories could be updated by mechanistic data and physicochemical property information are also described. This categorization approach may be used to form the basis of identifying candidates for data collection with related applications in QSAR development, read-across and hazard assessment.

## 1. Introduction

### 1.1. Background

Per- and Polyfluoroalkyl substances (PFAS) are a large class of human-made chemicals that have been manufactured and used in a variety of industries since the 1940s [1–3]. PFAS have been or are currently being synthesized for a myriad of different uses, including adhesives, stain resistant coatings for clothes or furniture and fire retardants. In addition to consumer and industrial applications, PFAS are being released into the environment during manufacturing and use [4]. PFAS and products containing them are regularly disposed of in landfills or incinerated, which can also lead to further release into soil, groundwater, and air [5,6]. They are also found in biosolids from wastewater treatment facilities which have been spread onto agricultural fields [7].

Characterizing the scope and scale of the ‘PFAS class’ has been challenging in the absence of a harmonized PFAS definition. Some publications have cited thousands of PFAS being in the environment (estimates range from 4700 [8] to greater than 10,000 [9]), but there is likely to be an increasing number given that analytical methods are continually being evolved to detect them. An Organisation for Economic Co-operation and Development (OECD) working group defined PFAS as ‘fluorinated substances that contain at least one fully fluorinated methyl or methylene carbon atom (without any H/Cl/Br/I atom attached to it); that is, any chemical with at least a perfluorinated methyl group (−CF_3_) or a perfluorinated methylene group (−CF_2_−)’ [8,10]. This broad OECD definition would make estimates of a few thousand PFAS too low; however, the OECD working group also acknowledges that a chemistry definition of PFAS does not necessarily equate to how PFAS should be assessed in terms of their hazard profile or to what extent subcategorizations of PFAS are appropriate depending on different legislative frameworks. Indeed, if the OECD definition were applied to a large inventory such as the US EPA’s Distributed Structure-Searchable (DSSTox) Database [11] estimates of the number of PFAS would be in the order of 30,000. For contrast, the PubChem Classification Browser has tagged over 7 million substances as meeting the OECD PFAS definition [12]. This would imply that any substance containing a CF_3_ would be classified as a “PFAS” even though it might fall within the purview of different regulatory frameworks. The US EPA’s Office of Pollution Prevention and Toxics (OPPT) recently finalized a structural definition of PFAS applicable to several Toxic Substances Control Act (TSCA) activities: the Significant New Use Rule (SNUR) on PFAS designated as inactive on the TSCA inventory [13], the TSCA Report and Record-keeping Requirements for Perfluoroalkyl and Polyfluoroalkyl Substances rule (referred to herein as the TSCA section 8(a)(7) rule) [14] and EPA’s recently released framework for assessing PFAS under TSCA’s New Chemicals activities [15]. For these TSCA actions, a PFAS is defined as ‘including at least one of three substructures: 1) R-(CF_2_)-CF(R’)R”, where both the CF_2_ and CF moieties are saturated carbons; 2) R-CF_2_OCF_2_-R’, where R and R’ can either be F, O, or saturated carbons; or 3) CF_3_C(CF_3_)R’R”, where R’ and R” can either be F or saturated carbons. This definition is narrower in scope than the OECD chemistry definition yet EPA estimates that it would still identify several thousand PFAS.

Of the many thousands of PFAS, few have been studied extensively in terms of their toxicity profile. Beyond a handful of closely-related perfluoroalkyl acids, perfluoroalkyl sulfonates, and perfluoroalkyl ethers (e.g., perfluoroocanoic acid (PFOA), perfluorooctane sulfonic acid (PFOS), and hexafluoropropylene oxide dimer acid, HFPO-DA), the vast majority of PFAS lack data to facilitate a robust characterization of their potential toxicity [16]. In an effort to address these data gaps, Congress directed EPA (15 USC 8962) to develop a process for prioritizing which PFAS or ‘class’ of PFAS should be subject to additional research efforts based on potential for human exposure, potential toxicity, and other available information. In response, the EPA published the EPA National PFAS Testing Strategy in October 2021 which describes EPA’s approach to developing categories of PFAS and identifying substances for further data collection efforts.

The notion of a ‘class’ underpins grouping approaches which includes the concept of developing categories to perform associated read-across. Rather than assessing each PFAS individually, closely related PFAS could be, in principle, grouped together into categories. Thus, in a category approach, not every PFAS needs to be tested for every single endpoint. Instead, the overall data for that category could potentially prove applicable to support a hazard assessment for other members of the category.

Grouping approaches have been in use in regulatory programmes for many years dating back to 1998 when guidance was developed by the EPA in support of the US High Production Volume (HPV) Challenge Program [17]. The concepts of grouping, categories and read-across are defined and extensively described in OECD’s grouping guidance document, last revised in 2014 [18] and presently undergoing revision. Moreover, the state of the art in read-across has also been described extensively in the literature; from workflows which outline the steps undertaken to develop category and analogue approaches through to the evaluation, justification and documentation of any read-across predictions made [19–22]. More recently the notion of enhancing structure-based groupings with new approach methods (NAMs) has also been an evolving topic. For example, of keen interest is the extent to which structural categories can be further justified by NAM data by providing a mechanistic underpinning [20–23]. NAMs are defined as any technology, methodology, approach, or combination that can provide information on chemical hazard and risk assessment without the use of animals, including *in silico*, *in chemico*, in vitro, and *ex vivo* approaches [24]. Of note, EPA has been leading a research programme to test a targeted set of ~150 PFAS through an array of different NAM approaches as part of a category approach [23,25–32].

This study describes the approach taken to further refine a relevant PFAS landscape to EPA from which an initial set of structural categories were derived. The work here is a continuation of the initial categorization efforts described in the EPA National PFAS Testing Strategy. For the categories developed, data gaps were assessed to help identify which categories were particularly data poor (e.g., lacking relevant repeated dose toxicity data) and/or associated with known exposures and therefore would benefit from data collection or new test data generation (using both NAMs or traditional approaches) to better characterize the category as a whole. The aims of this manuscript are as follows:

1. Summarize the process of constructing a PFAS landscape;
2. Profile the PFAS landscape to assign substances into broad structural categories in combination with chain length;
3. Evaluate the degree of structural similarity within each category and determine which categories needed to be further subset to maximize their structural similarity whilst maintaining a pragmatic total number of categories;
4. Facilitate the identification of potential candidate PFAS for data collection by capturing additional considerations such as availability of a known manufacturer/importer (who would be responsible for conducting testing via TSCA); EPA Agency and/or State priorities, environmental monitoring information and structural diversity within the category;
5. Evaluate the categories based on their predicted physical state and physicochemical properties (a context of evaluating the similarity within the category and informing on potential technical limitations for testing);
6. Consider the utility of the structural categories developed in performing read-across, as well as refinements such as incorporating mechanistic and toxicokinetic data derived from NAMs. The mechanistic insights derived from EPA’s parallel research effort on selected PFAS offer potential opportunities to refine the structurally-based categories developed.
7. Evaluate the feasibility of operationalizing the structural categories so that new PFAS can be profiled and assigned into one of the terminal categories developed.

## 2. Methods

### 2.1. Defining the PFAS landscape

To define the PFAS landscape for the purpose of this study, the DSSTox database [11,33] was searched using a series of structure-based queries that reflected the PFAS structural definition described earlier (see Section 1.1). DSSTox forms the basis of the EPA CompTox Chemicals Dashboard (referred to herein as the Dashboard) [11,33] and comprises 1,218,248 substances (at the time of writing, May 2024, https://comptox.epa.gov/dashboard/). As a result of the search, 13,054 substances were identified as forming the initial PFAS landscape for this study. This landscape is available as a list published on the Dashboard at PFAS8a7v3. This set was then cross referenced with the TSCA inventory (see Section 2.4.1) to identify matches. The TSCA inventory is the list of chemical substances in commerce (manufactured, processed or imported) in the US since January 1975 that do not qualify for an exemption or exclusion under TSCA (TSCA inventory; Section 2.10.1). Note that EPA maintains two TSCA inventories – one that is publicly available and another that contains confidential business information (CBI) and is not publicly available. Those substances reported to EPA as in commerce since June 2006 are designated as “active” on the TSCA inventory. For each of the PFAS (active and inactive) listed on the publicly available TSCA inventory, degradation products were simulated using the biodegradation model, Catalogic 301C v13.18 within the commercial software tool, OASIS Catalogic v5.16.1.10 (University As Zlatarov, Laboratory of Mathematical Chemistry, Bourgas, Bulgaria; http://oasis-lmc.org/). The intent was to enrich the landscape for PFAS likely to be found in the environment that originated from substances in commerce. The set of PFAS degradation products (2484) for the parent TSCA substances were added to the initial landscape such that the final PFAS landscape used in this study comprised 15,538 substances. Note only degradation products meeting the PFAS definition were considered. Chemicals were represented by unique DSSTox Substance Identifiers (DTXSID) [11], Simplified Molecular-Input-Line-Entry System (SMILES) (https://www.daylight.com/dayhtml/doc/theory/theory.smiles.html), chemical names and CAS Registry Numbers (CASRN). International Chemical Identifier keys (InChIKeys), (hashed InChI) [34] were used as identifiers for the degradation products. Chemical substances in the DSSTox database have been curated and standardized to ensure correctness in chemical structure as well as their associations to chemical names and other identifiers such as CASRN. Examples of this curation include checking for errors and mismatches in chemical structure formats and mapping to identifiers, as well as structure validation and/or standardization issues such as hyper-valency, tautomerism, etc. [11].

### 2.2. Biodegradation potential

Biodegradation predictions were made for PFAS in the landscape that were on the TSCA inventory using the Catalogic 301C v13.18 model within the commercial software tool, OASIS Catalogic v5.16.1.10. The biodegradation Catalogic 301C model simulates aerobic biodegradation under Ministry of International Trade and Industry, Japan (MITI) I (OECD 301C) test conditions. The modelled endpoint is the percentage of theoretical biological oxygen demand (BOD) on day 28. The underlying training set for the model comprises BOD data for 2618 substances – 745 of these were collected from the MITI I database and 804 were provided by National Institute of Technology and Evaluation (NITE), Japan. A further 1069 substances that were proprietary were provided by NITE, Japan. The training set includes 797 readily biodegradable and 1821 not readily biodegradable substances. In addition to BOD data, a second database underpinning the model comprised pathways for 845 organic substances, documented pathways for 649 chemicals were collected from the primary and secondary literature whereas pathways for 196 proprietary substances were provided by NITE, Japan. In brief, the Catalogic model comprises a metabolic simulator and an endpoint model. The microbial metabolism is simulated by a rule-based approach based on a set of hierarchically ordered transformations and a system of rules controlling the application of these transformations. Recursive application of the transformations allows for the simulation of metabolism and generation of biodegradation pathways. Calculation of the modelled endpoint is based on the simulated metabolic tree and the material balance of transformations used to build the tree. Predictions were made for all PFAS in the landscape that were on the non-confidential TSCA inventory (see Section 2.4.1 for more details). Prediction results containing the list of simulated metabolites (as SMILES) along with their parent DTXSID identifiers were exported as a text file. Prediction results were then processed in the following manner:

1. DTXSID identifiers were extracted for each parent substance and mapped to each metabolite. This ensured for a given parent, all metabolites could be readily associated with its corresponding parent substance.
2. A new identifier was then created for the metabolites based on the parent DTXSID identifier. That is to say, the first listed metabolite simulated for parent DTXSID9065256 would be tagged as DTXSID9065256_m_1 and so on.
3. InChIKeys were then generated for all SMILES, parents and simulated metabolites. Use of InChIKeys provided an unambiguous means of structurally representing the substance (rather than using SMILES that are potentially non-unique) and enabled subsequent associations to be derived between substances. The processed results were saved for subsequent analysis.

Many degradation products were found to be common across parent substances. Grouping by InChIKeys created a set of unique degradation products. These were filtered to remove non PFAS degradation products or those not meeting the PFAS definition. A final step involved cross matching the degradates against the starting landscape to remove any duplicate entries i.e. if a degradate was a substance already captured. These steps resulted in a set of 2484 degradation products that were then added to the starting landscape of 13,054 substances.

To explore the coverage and relevance of the MITI training set (the non-proprietary portion) within the Catalogic 301C model relative to the PFAS on the TSCA inventory substances, a comparison was performed to assess the overlap in structural space as characterized by Morgan chemical fingerprints [35] (see Section 2.3.4 for details on chemical fingerprint generation). In the latter case, this structural space was projected onto a 2-dimensional (2D) scatterplot (see Fig. S1) using a Uniform Manifold Approximation and Projection (UMAP) to facilitate visualization [36]. This is a dimensionality reduction technique that assumes available data samples are evenly distributed across a topological space (manifold) which can be approximated from these finite data samples and mapped to a lower dimensional space. In essence, UMAP learns the manifold in the high dimensional space, in this case, these are the 1024 chemical fingerprints and aims to find a 2D representation of the same manifold. The Catalogic model also provided an indication of whether any of the PFAS profiled were part of the training set as well as whether they were within the structural domain of applicability [37].

### 2.3. Profiling PFAS into structural categories

**Fig. 1.**
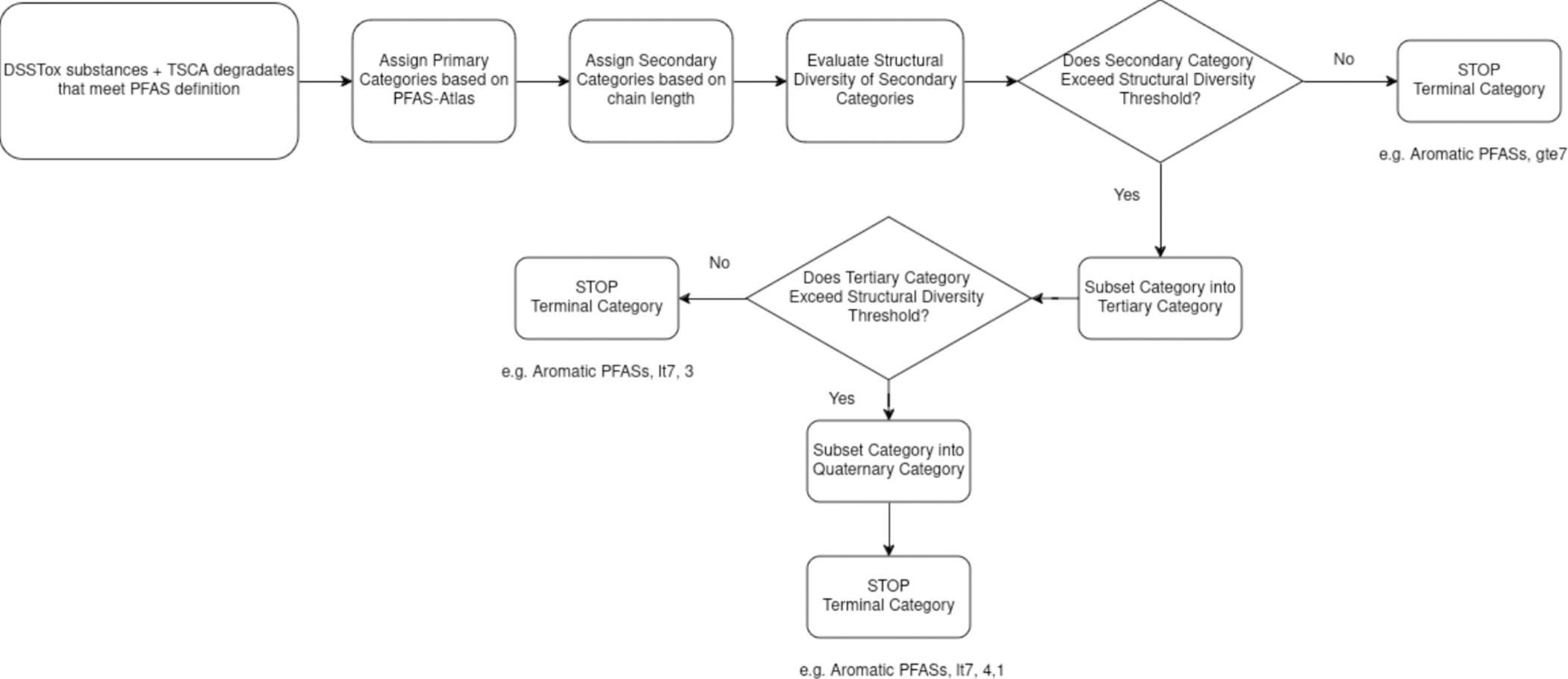
Conceptual workflow for generating PFAS structural categories.

This study aimed to develop a hierarchy of PFAS categories starting with a handful of large, diverse categories that could be subcategorized into more structurally similar categories based on other considerations (e.g., chain length and chemical fingerprints). The conceptual workflow for creating the PFAS structural categories is summarized in Fig. 1 and the details of each step are described in turn.

#### 2.3.1. Primary structural categories

Primary categories were derived by profiling the PFAS landscape of 15,538 substances through the PFAS subgroup classification tool developed by Su et al. [38] called PFAS-Atlas. As described in Su et al. [38], PFAS can be classified into one of at least four broad classes:

- Perfluoroalkyl acids (PFAA)
- PFAA precursors
- Polyfluoroalkyl acids
- Other PFAS

In practice, when substances are batch processed by PFAS-Atlas, a first class and second class are assigned. The PFAS-Atlas first class corresponds to the 4 primary categories described above but additionally delineates linear substances from cyclic substances to create 8 primary categories. Any substance that is not a PFAS by the structural definitions within PFAS-Atlas are designated as ‘Not PFAS’. The PFAS-Atlas second class subdivides each of the first classes further, for example in the PFAA precursors categories, hydrofluoroethers would be classified separately from semi-fluorinated alkenes or perfluoroalkane sulfonyl fluorides (PASFs). For this study, a hybrid approach was used taking into account membership size. If the number of substances in a PFAS-Atlas first class designation exceeded 300 substances, its second class designation was used. This was performed to limit the number of primary structural categories, and avoid large membership sizes such as too many substances falling into the ‘Other PFAS’ category. The 300 membership threshold was chosen after manual inspection of the membership counts following substance assignment into the PFAS-Atlas first and second class designations. The PFAS-Atlas first class designation was used as the initial primary category assignment for PFAAs, whereas a handful of second class assignments were used for PFAA precursors and Polyfluoroalkyl acids. The PFAS-Atlas second class designation was used in lieu of “Other PFAS” except when the number of substances were very low. The PFAS-Atlas classification tree is an update of the original PFAS-Map database framework developed by some of the same authors [39] (and which had been used in the original NTS) but is more closely aligned with the OECD Terminology 2021 guidance [8] with some modifications.

The PFAS landscape was also processed through the OPEn structure–activity/property Relationship App (OPERA) v2.9 tool [40] https://github.com/kmansouri/OPERA to derive QSAR-READY SMILES and selected physicochemical property predictions (as discussed in Section 4.3). QSAR-READY SMILES are standardized SMILES where salts and stereochemistry are removed. QSAR-READY SMILES were used to faciliate the processing of substances through PFAS-Atlas. Substances without QSAR-READY SMILES or which could not be computationally resolved were assigned as “unclassified”. Substances assigned as “Not PFAS” by PFAS-Atlas were also re-assigned as “unclassified”.

The category assignments used in this study are captured in Table 1.

#### 2.3.2. Secondary structural categories

It is hypothesized that the length of the contiguous fluorinated carbon chain influences differences in toxicity as well as the length of time the chemical spends in the body and environment. This supposition draws from experiences with PFAAs [41,42]. Due to the potential importance of chain length in the toxicity, persistence and bioaccumulation of PFAS, secondary structural categories were defined using a carbon chain length threshold.

##### 2.3.2.1. Chain length determination.

The maximum number of contiguous CF_2_ groups in a chain was determined for all 15,538 substances. This was achieved by iterating through a range of CF_2_ chain lengths (from 1 to 30) for each substance in turn and determining its longest chain length. For instance, Perfluorosebacamidine [DTXSID40380015] contains 8 contiguous CF_2_ units; hence, its chain length was denoted as 8. PFOA [DTXSID8031865] had a maximum chain length of 7 whereas PFOS [DTXSID3031864] had a maximum chain length of 8. For PFOA, although there are 8 carbons in its backbone, the 8th is part of the carboxyl group whereas in PFOS, there are 8 CF_2_ groups plus the sulfonate group.

For the current analysis, the chain length threshold was set at 7 (>=7 vs < 7) as representative of a “long chain” PFAS. The chain length threshold is broadly consistent with the EPA’s 2009 PFAS action plan. A PFAS with a maximum number of contiguous CF_2_ number greater than or equal to 7 was denoted “gte7”. Using this threshold, both PFOS and PFOA would be assigned to the “gte7” secondary category. A PFAS with a maximum number of contiguous CF_2_ groups less than 7 was denoted “lt7”. Defining chain lengths for PFAS with non-contiguous chains or branching is less straightforward but has been evaluated in more detail by Richard et al. [43,44] through the development of new PFAS specific chemical fingerprints, so-named PFAS ToxPrints, as an extension of the logic used to develop the original ToxPrints that had been defined for a broader chemistry [45]. A secondary category was thus denoted by its PFAS-Atlas assignment, akin to the primary OECD structural classification and a carbon chain length threshold e.g., 2,2,3,3,4,4,5,5,6,6,7,7,8,8,9,9,9-Heptadecafluoro-N,N-diphenylnonanamide [DTXSID90896196] would thus be described as belonging to the “Aromatic PFASs, gte7” secondary category (see Fig. 1).

#### 2.3.3. Derivation of terminal structural categories

The underlying motivation for the study was to identify categories that would balance maximizing structural similarity that could permit read-across within those categories versus pragmatism in terms of total number of categories. Too many categories with very few substances renders the approach less generalizable, too few categories could result in extrapolating between substances that were not sufficiently similar. To that end, an objective threshold was needed to determine how granular categories needed to be to manage this trade-off and ensure that the categorization was actionable. An objective threshold was developed, described in Section 2.3.6, that compared structural similarity within a category relative to the structural similarity between different categories.

#### 2.3.4. Chemical fingerprints

Morgan chemical fingerprints [35] were calculated for all substances within each secondary category using the open-source Python library RDkit [46], with a radius of 3 and a bit-length of 1024. The fingerprint data was represented as bit vectors where presence of structural features were denoted by 1 and absence by 0. These fingerprint (FP) files were stored for subsequent processing. Morgan fingerprints also known as Extended Connectivity Fingerprints (ECFPs) are widely used in machine learning applications for cheminformatics [47] especially when ranking diverse structures by similarity. These circular fingerprints map the molecular environment of every atom.

#### 2.3.5. Chemical similarity

Pairwise distance matrices were calculated for each secondary category. These were generated by using the chemical fingerprint files as inputs and computing the Jaccard distance for each pair of substances. The Jaccard distance captures the proportion of FP bits between 2 substances that differ [48]. The Jaccard distance ranges from 0 to 1 where 0 would indicate zero distance (or high similarity) and 1 would indicate high distance (or low similarity). Distance matrices were computed for all secondary categories and stored for subsequent processing. These are referred to as ‘within category’ distance matrices in Section 2.3.6.

#### 2.3.6. Objective distance threshold

The rationale underpinning the objective distance threshold was based on the expectation that the variance in the distribution of the pairwise distances for each secondary category representing the ‘within category’ similarity would be lower than distributions of the pairwise distances between different secondary categories (‘between category’). The ‘within category’ distances had already been computed as described in Section 2.3.5.

‘Between category’ combinations aimed to identify categories that did not share the same primary category root. A list of all possible binary combinations of secondary categories was created using the names of the secondary categories, “Aromatic PFASs, lt7” and “PFAA precursors, gte7” is an example of such a binary combination. These were then filtered to remove secondary categories that shared the same primary category root (i.e., a combination such as “PFAAs, lt7” and “PFAAs, gte7” would be excluded from consideration as a ‘between category’). Chemical fingerprint datasets for each binary combination were created by combining the secondary category chemical fingerprint datasets. Pairwise distance matrices were then derived for the combined category set. These matrices were filtered to retain only the pairwise distances between the starting secondary categories.

The empirical cumulative distribution functions (ECDFs) of the pairwise distances were calculated for each secondary category (see Fig. S2 for a plot of the ECDFs). ECDFs were also derived for the ‘between category’ combinations (see Fig. S3 for a plot of the first 10 ECDFs). The ECDFs permitted a visual inspection of the range of the pairwise distances across all secondary categories as well as across all ‘between category’ combinations. Based on visual inspection of the ECDFs, the median value for each distribution was selected as the summary metric.

Probability density functions of median values from all within and between secondary categories were plotted to explore their overlap. The 15^th^ percentile of the ‘between categories’ distribution was selected, by reference to the density plot, as the threshold to determine whether a secondary category merited further subcategorization. A secondary category was only subcategorized if the median of its ‘within category’ pairwise distance distribution exceeded this threshold.

#### 2.3.7. Deriving terminal categories

Secondary categories that exceeded the threshold were subcategorized using agglomerative hierarchical clustering. The condensed form of the pairwise distance matrix computed for each secondary category that exceeded the threshold was used as an input into a hierarchical clustering using Ward’s method [49]. Ward’s method is a criterion that minimizes the total within-cluster variance. For each secondary category, the dendrogram was plotted and the number of first-generation clusters was set as the maximum cluster number. Clusters were labelled as 1,2,3 etc. Each of the clustering results were combined into one table which was then merged with the starting table of primary and secondary categories.

The next generation of categories, quaternary categories, would then be processed in the same manner to determine whether any exceeded the objective threshold and needed to be subcategorized further as already described. In practice, a maximum of two generations of subcategorizations were performed, with the expectation that this would balance the structural similarity within the category relative to total number of terminal categories.

Secondary categories or tertiary categories which did not exceed the threshold were ultimately denoted as the terminal category. Thus, a terminal category could be tagged as “Aromatic PFASs, gte7”, effectively a secondary category, or could be tagged as “Aromatic PFASs, lt7, 3”, a tertiary category or following two iterations of subcategorization would be tagged as “Aromatic PFASs, lt7, 4, 1” (see Fig. 1). Note: in the data files and figures, terminal categories without one or two iterations of subcategorization are denoted as “Aromatic PFASs, gte7, nan, nan” or “Aromatic PFASs, lt7, 3, nan” where “nan” represents a null value.

#### 2.3.8. Identification of centroid substances

For each terminal category, a single substance was identified that was nominally representative of the category. This substance was the computed centroid calculated from the Jaccard pairwise distance matrices (see Section 2.3.5). The sum of the pairwise distances across all substances for a given structural category was computed and the substance with the minimum value was denoted as the centroid (i.e., this substance would have the lowest distance from all other category members). Technically, this calculation gives rise to the medoid of a cluster. However, for the purposes of this analysis and for consistency with the NTS, the term centroid is used to denote it as the ‘central’ substance within the category. Distances of all category members relative to the centroid substance were also computed.

#### 2.3.9. Identification of additional representative substances

Since a number of the terminal categories were large in size (e.g. greater than 300 members), a single substance would be potentially insufficient to both characterize the category and its potential hazard profile. In an effort to address this limitation, the MaxMinPicker approach, as implemented within the RDKit Python library, was applied to identify additional substances which would in turn capture the breadth and diversity of each terminal category [50]. The MaxMin approach is a well-established algorithm for dissimilarity-based compound selection that has been applied in drug discovery for many years. The reader is referred to Snarey et al. [51] for a comparison of the different algorithms. The MaxMinPicker approach proceeds as follows:

1. Molecular descriptors are generated for all substances. In this case, the Morgan fingerprints calculated for all substances within a terminal category represented the candidate pool whereas the pre-computed centroid equated to the initial seed.
2. From the substances in the terminal category, the substance that had the maximum value for its minimum distance to the picked set (initially this would be just the centroid) would then be identified. This substance would be the most distant one to those already picked so it would be transferred to the ‘picked set’ (now centroid + 1).
3. An iteration back to step 2 would then be performed until the desired number of substances were picked

The MaxMinPicker was applied to all terminal categories containing more than 5 members to identify the next 3 most diverse substances within a category (centroid + up to 3 additional substances). The intention of identifying additional diverse substances was to help bound the domain of the structural category. The identification of 3 most diverse substances was chosen out of convenience to provide an actionable number of additional substances.

A systematic evaluation of the relationship between the number of diverse substances that could be identified relative to the structural diversity within each terminal category was also undertaken. This was performed as follows, first the ranked order by diversity of all members within a terminal category was computed. Then the pairwise distance matrices derived in Section 2.3.5 were filtered by the diverse substances, starting from the centroid, centroid plus first diverse chemical through to the complete set of category members. At each step the mean minimum distance was recorded. This enabled the construction of a matrix to capture the mean of the minimum pairwise distances relative to the number of diverse chemicals selected. The normalized cumulative sum of all the mean minimum distances was then computed. This provided a means of evaluating the proportion of structural diversity that was captured as a function of the number of MaxMin substances identified. This calculation provides for two objective assessments namely:

1. The amount of structural diversity captured by the 3 diverse picks originally identified; and
2. the number of diverse substances that would need to be identified (if practical resources were not a limiting factor) to capture a specified level of structural diversity. For example, how many substances would need to be identified if capturing a specific percentage of the structural diversity within a terminal category was desired, 80% is presented here merely for illustrative purposes.

### 2.4. Facilitating the identification of potential candidates for data collection

To facilitate the identification of potential candidate PFAS for data collection, availability of a known manufacturer/importer, EPA Agency and/or State priorities, environmental monitoring information were evaluated as additional considerations. These are described in turn.

#### 2.4.1. Qualitative exposure and release designations

Several qualitative designations were added to the landscape to identify substances for which exposure could be plausible, including their TSCA inventory status, production volumes per TSCA’s Chemical Data Reporting (CDR) rule, State/EPA Region priorities, as well as physical state and physicochemical properties.

The non-confidential (Non-CBI) TSCA Inventory active and inactive lists were downloaded from the Dashboard (see TSCA_ACTIVE_NCTI_0224,TSCA_INACTIVE_NCTI_0224) and combined into one large set. Substances within this inventory included both Chemical Abstract Service (CAS) Registry Number, Chemical Abstracts (CA) Index Name, and DSSTox substance identifier (DTXSID). These were matched by the DTXSID identifiers already captured in the PFAS landscape. Substances were tagged as ‘inactive’, ‘active’ or ‘unclassified’. Note the predicted degradation products of substances tagged as either “inactive” or “active” had been used to augment the PFAS landscape as already described in Section 2.1.

The 2020 CDR data was downloaded from the public EPA web address (https://www.epa.gov/chemical-data-reporting/access-cdr-data). The CDR data comprises information for a set of 8660 substances. DTXSID identifiers were available for 8017 of these substances when using the Batch search functionality within the Dashboard. A tag was created for CDR2020 status if a PFAS had a 2020 CDR record. National Aggregated Production Volume (National Agg PV) data was also extracted to highlight how this was distributed across primary categories. Since some of the production volume (PV) data was numeric and some represented in numeric ranges, the PV data was summarized into one of 10 different ranges (<25,000 lbs, 25,000–<100,000 lbs, 100,000–<500,000 lbs, 500,000–<1,000,000 lbs, <1,000,000 lbs, 1,000,000 – <10,000,000 lbs, 1,000,000–<20,000,000 lbs, 20,000,000–<100,000,000 lbs, 50,000,000-<100,000,000 lbs, 100,000,000–<1,000,000,000 lbs).

Various EPA Regions or States have identified PFAS of interest based on validated analytical methods or for environmental monitoring purposes. The data sources captured as part of the EPA’s PFAS Analytic Tools website (https://echo.epa.gov/trends/pfas-tools#data) were used to construct lists of such PFAS. The specific data sources were Discharge Monitoring Data, Drinking Water (State) Data, Drinking Water (Unregulated Contaminant Monitoring Rule (UCMR)) Data, Environmental Media Data, Production Data, Toxics Release Inventory (TRI) Data – Waste Managed, TRI Data – On-Site, TRI Data – Off-Site and Production Data (all accessed 7th April 2024). Discharge Monitoring data is collected by virtue of the National Pollutant Elimination System permit. Drinking Water Data comprises UCMR and State level monitoring data. Environmental Media data comprises ambient sampling data reported by federal, state, tribal and local governments, academic and non-governmental organizations, and individuals that are submitted to the Water Quality Portal (WQP). Production data entails information reported under the Chemical Data Reporting (CDR) Rule under TSCA. TRI tracks the management of certain toxic chemicals that may pose a threat to human health or the environment by more than 21,000 facilities throughout the US and its territories. The National Defense Authorization Act of Fiscal year 2020 (NDAA) added certain PFAS to the TRI list and provided a framework for the ongoing listing of additional PFAS.

Identifiers were extracted from these source files and searched against the Dashboard to map to DTXSID records. The set of identifiers (Names and CASRN) within the entire PFAS landscape were also queried against PubMed, the National Library of Medicine’s citation index for biomedical literature, to determine whether studies for a substance might have been reported in the literature. The article counts were obtained using the same queries as used within the Abstract Sifter v7.5 [52].

Expected routes of exposure and presence in environmental media are dependent on the physical state and physicochemical properties. Physicochemical properties were predicted using the open-source OPERA v2.9 tool [40] for all substances with QSAR-READY SMILES (as discussed in Section 2.3.1). The properties predicted were melting point, boiling point, Henry’s Law constant (HLC), water solubility and vapour pressure. Physical state was predicted at 25 deg C using the predicted values of melting point and boiling point. Gases had boiling points less than 25 deg C, solids had melting points greater than or equal to 25 deg C and liquids had melting points less than 25 deg C and boiling points greater than or equal to 25 deg C. These are the guiding principles underpinning the EPA’s Sustainable Futures Framework guidance (see Interpretative Guidance Document). Whilst physical properties are continuously distributed, and cutoff values are necessarily arbitrary, there is utility in grouping substances into broad categories as a way to acknowledge the practicalities of testing and human exposure under “typical” (i.e. room temperature and atmospheric pressure) conditions. A water solubility threshold of 0.5 mg/L was used to denote whether a substance was soluble/insoluble whereas a vapour pressure threshold of 75 mmHg determined volatility and a HLC threshold of 0.1 atm m^3^/mol highly volatile. Based on these properties, each substance was assigned into 1 of 4 “physical state and physicochemical designations” (from A-D). Designation A covered substances that were insoluble solids, designation B captured both soluble solids and soluble non-volatile liquids, whereas C tagged soluble volatile liquids/insoluble liquids and soluble gases. Designation D assigned substances as insoluble gases or highly volatile gases. Substances that could not be assigned into one of these 4 designations were tagged as ‘not determined’. For each of the terminal structural categories, Morgan fingerprint representations were projected into two dimensions using UMAP to facilitate visualization [36]. The projections were plotted as 2D kernel density distributions overlaid with physical state and physicochemical designation information to help explore the extent to which members were assigned to the same designation and therefore had a consistent profile across a given terminal category.

Each of these respective qualitative designations were then matched to the PFAS landscape to provide another attribute for consideration when identifying potential candidates for data collection.

#### 2.4.2. Constrained PFAS landscape

One of the limitations of the identification of centroids and additional diverse substances was that they might yet not yield feasible candidates for data collection due to the lack of assignable manufacturer/importer. This was articulated as a potential challenge in the National PFAS Testing Strategy. To address this practical constraint, the same process of computing centroids, identifying additional diverse substances and evaluating their structural diversity coverage was also performed using the terminal categories as a basis as described in Section 2.3.7 but constraining the landscape to only those substances on the public TSCA inventory and specifically those substances that were actives on the public TSCA inventory. Constraining the landscape would allow identification of substances for data collection that were already in commerce and/or could be more readily procured.

### 2.5. Evaluation of variance of in vivo toxicity within terminal categories

Ultimately, read-across of data within categories could be performed such that the hazard profile of the category is adequate without needing to test a significant number of category members. To evaluate the feasibility of performing read-across within the terminal categories derived, an exploration of the distribution of *in vivo* points of departure (PODs) within and across terminal categories was performed for the oral route of exposure.

#### 2.5.1. Variance of in vivo PODs across and within terminal categories

From ToxValDB version 9.5, the Toxicity Values Database, all studies where ‘oral’ was the route of exposure were extracted. Only records where a point of departure (POD) was reported as a NOEL, NOAEL, NEL, NOAEC, LOAEL, LOEL, LOAEC, LEL and where the dose units were expressed as mg/kg-bw/day or mg/kg were retrieved. Study types were also restricted to the following: ‘short-term’, ‘subchronic’, ‘chronic’, ‘developmental’, ‘reproduction’, ‘reproduction developmental’, ‘28-day’ as captured in the ‘study type’ field within the database. Species were standardized into one of ‘rat’, ‘mouse’, ‘rabbit’, ‘dog’, ‘hamster’ or ‘guinea pig’. Effect levels were harmonized consistent with the approach taken by Aurisano et al. [53] where non-cancer effects vs. reproductive/developmental effects were processed separately. Records with sub-acute or sub-chronic as the study type were extrapolated to chronic using a subchronic-to-chronic factor of 2 and a subacute-to-chronic factor of 5^2^. LOAEL as effect level types were extrapolated to NOAELs by dividing by an extrapolation factor of 3. Effect levels for all records were extrapolated to humans by dividing reported effect values by conversion factors based on the average body of weight of humans relative to the average body weight of the test species. NOAELs were extrapolated to human equivalent Benchmark Dose (BMDh) values based on assigned conceptual models depending on the critical effect reported. The calculated BMDh was based on the mean of 2 assigned conceptual models. In Aurisano et al. [53], the 25^th^ percentile of the fitted log-normal distributed (using the mean BMD and standard deviation (sd) BMD) was calculated to derive a POD per substance. The sd was set to the median sd of all records in cases where the number of study records was less than 5.

The derived BMDh values were then merged with the PFAS substances from the landscape. The summary values provided an estimate of the POD for each substance and the expected level of variation across and within categories. Box and whisker plots were created to reflect the distribution of the PODs across the terminal categories for general non-cancer and repro/developmental effects for the oral route of exposure. Strip plots were overlaid to show the variation of chain length across a given terminal category for general non-cancer effects.

### 2.6. Qualitative mechanistic and toxicokinetic designations

A summary of the NAM testing being undertaken for ~150 PFAS was described in Patlewicz et al. [23]. See Houck et al. [26] for results from various nuclear receptor and oxidative stress targeted assays, Houck et al. [27] for 12 human primary cell-based assay models of pathophysiology including immunosuppression, Carstens et al. [25] for the developmental neurotoxicity assays, Degitz et al. [32] for the thyroid pathway assays and, for toxicokinetic information, Smeltz et al. [29] and Kreutz et al. [28]. The manuscript for the remaining data stream (zebrafish developmental toxicity) is in under internal review (Britton et al., *in prep*).

In addition to the NAM testing, a quality control (QC) evaluation of the chemical stock solutions was undertaken to confirm PFAS analyte presence and stability [30]. This evaluation was warranted given recent reports of certain PFAS degrading in the aprotic solvent dimethyl sulfoxide (DMSO), readily used as the solvent of choice in HTS [54,55]. Two hundred and five PFAS selected based on criteria described in Patlewicz et al. [23] were evaluated using low resolution tandem mass spectrometric detection strategies to confirm presence of intended analyte, evaluate analyte stability and presence of isomers, and verify stock concentrations for a subset for which commercially available verified standards were available. Ultimately 57 PFAS failed QC evaluation, with three exhibiting degradation in DMSO and the remainder not detected as present, likely due to volatilization. The pass/fail scores and informational flags as described in Smeltz et al. [30], and can be downloaded from the following figshare url, https://epa.figshare.com/articles/dataset/Chemistry_Dashboard_Data_Analytical_QC_for_PFAS/22118099.

For each of the NAM data streams, substances were tagged with a qualitative flag to indicate the class of mechanistic information that could be derived from the associated assay outcome (e.g., estrogen receptor activity from a nuclear receptor assay) and an expert-derived qualitative level of confidence associated with the outcome (high confidence of activity, medium confidence of activity or low concern). Only NAM results from substances that passed QC were carried forward. These flags were considered as an additional line of evidence to determine whether a terminal category might merit being split based on its mechanistic or toxicokinetic information or to inform what types of higher order testing might be most impactful for a given substance drawn from said terminal category. The derivation of the flags are described in more detail in Judson et al. *in prep*. Confidence scores across the NAM flags were standardized as appropriate to facilitate visualizations across data streams. Each flag could take on one of three values, low concern, medium or high confidence, color coded as blue, yellow and red. The immune flag was the exception. It was binary in nature and only gave rise to a low concern and medium confidence value. The flag categories are summarized below in Table 2. One final parameter computed was a TK half-life bin score using the machine learning model developed by Dawson et al. [56]. Predictions were scored from 1 to 4 where 1 signified a half-life ≤ 12h, 2 = 12h – 1 week, 3 = 1 week – 2 months, and 4 ≥ 2 months. Predictions were generated for humans assuming a oral dosing regimen and aggregated by maximum half-life score so that there was a single prediction per substance.

Qualitative observations of the consistency of the various flags across all the tested substances and within terminal categories were made. The Fisher’s exact test was used to compute an odds ratio and associated p-value for each PFAS ToxPrint [44] relative to a NAM flag that had been converted into a binary scale. This enrichment analysis was comparable with the methodology discussed in Wang et al. [57]. A PFAS ToxPrint was considered enriched if it had an odds ratio greater than or equal to 3, an one-sided Fishers exact p-value less than 0.05 (probability value of the odds ratio being greater than 1) and the number of true positives equal or greater than 3. The set of ‘enriched’ PFAS ToxPrints were then used to profile the entire PFAS landscape to assign potential predicted NAM flags. Comparisons were made of the actual NAM flags and their predictions to evaluate performance metrics (sensitivity and specificity). TK half-life predictions were generated for all substances and the outcomes binarized where substances with a bin category of 4 were assigned a 1, and any other bin category lower was assigned a 0.

### 2.7. Operationalizing the terminal categories for re-use

In the absence of a model to predict the terminal category, the categorization would need to be re-run for each new set of PFAS. To operationalize the terminal categories for practical use, a machine learning approach was used to develop a model that could be used to profile a new PFAS and assign it to its most likely terminal category. A random forest classifier (RFC) as implemented in the python library scikit-learn [58] was used to predict assignment of substances into one of the final terminal categories developed. Morgan fingerprints generated earlier (as discussed in Section 2.3.4) in conjunction with primary category and chain length were combined with the final terminal category names for all substances. Terminal categories with less than 10 members were aggregated together into one miscellaneous category. The dataset was then split into a training 80% and test 20% split using a random stratification approach based on the terminal category labels. A dummy classifier was applied first to establish a baseline. Then an intial RFC with default settings was assessed within a 5-fold stratified cross validation (CV) procedure to evaluate initial performance using balanced accuracy as a metric. This was performed using a pipeline within scikit-learn where the primary category names were treated as category features and passed into an OrdinalEncoder whereas chain length and Morgan fingerprints were first standardized before being passed to the RFC. A randomized search was then undertaken as part of a nested 5-fold CV to identify the best parameters and evaluate test CV performance. The resulting model was then applied to the test set that had been held out to evaluate performance. Finally, the model was refitted to the entire dataset.

## 3. Data analysis software and code

Data processing was conducted using the Anaconda distribution of Python 3.9 and associated libraries. Jupyter notebooks are available at https://github.com/patlewig/nts_pfas. Datasets supporting the manuscript are accessible at https://doi.org/10.23645/epacomptox.26524327.

## 4. Results and discussion

### 4.1. Primary and secondary structural categories

The PFAS landscape following application of the TSCA section 8(a) (7) rule to DSSTox resulted in a dataset comprising 13,054 substances plus 2484 degradation products for a total of 15,538.

Minimal structural overlap was found between the 1549 accessible training set substances (of which 1429 substances could be resolved into structures) from the Catalogic model and the TSCA inventory substances. Fig. S1 depicts a UMAP plot for the MITI training set substances relative to the TSCA substances using Morgan chemical fingerprints as inputs. There were 12 TSCA substances of the dataset that were part of the training set. Only 29% of the PFAS TSCA substances were tagged as being within the structural domain of the model. In view of this, the degradation products simulated (comprising 16% of the landscape) should be interpreted with caution until additional experimental data are collected and new models developed.

**Fig. 2.**
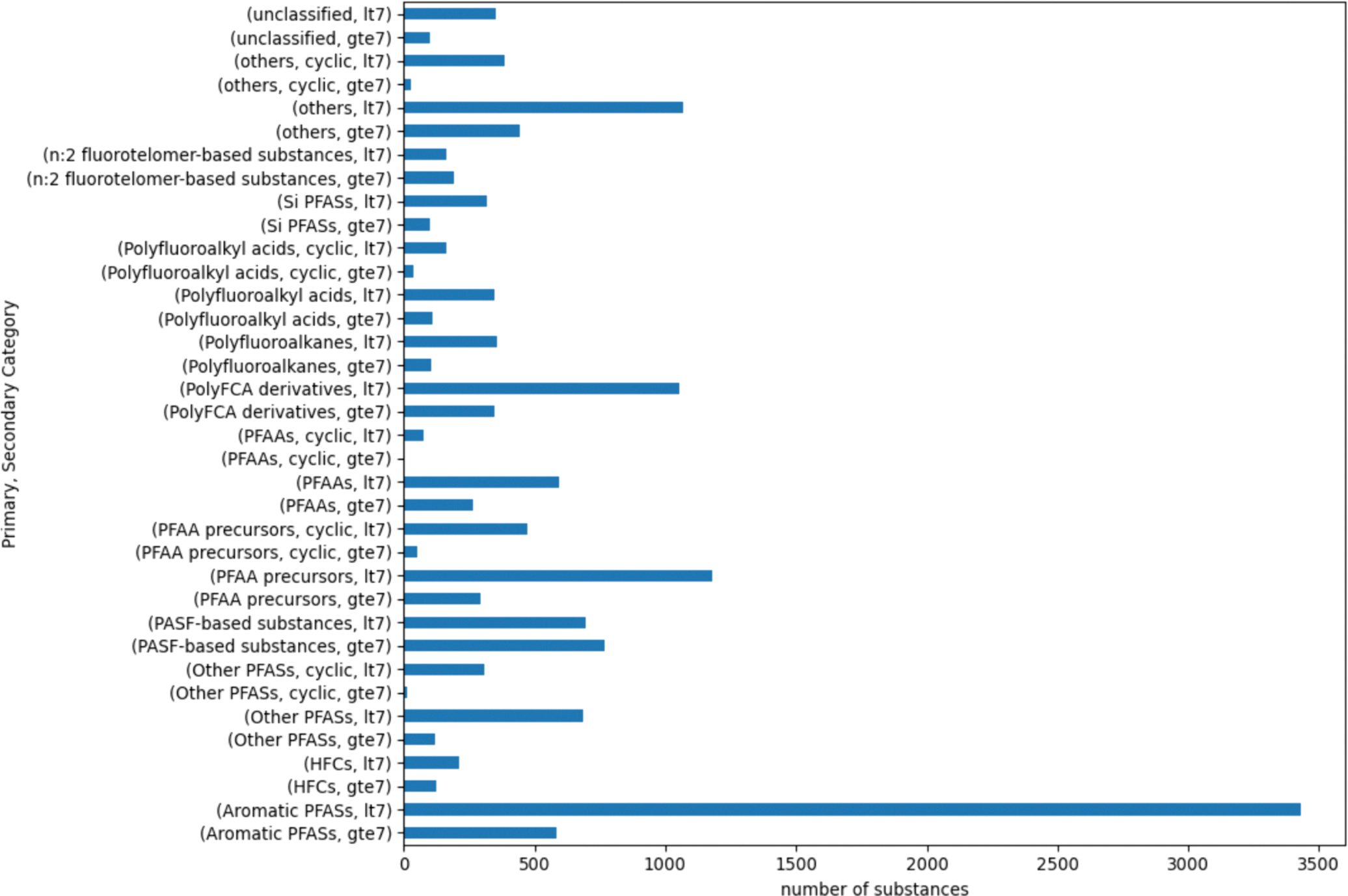
Bar chart showing the number of substances within each secondary category, ordered by primary category root. Methods to define primary and secondary categories are outlined in Sections 2.31 and 2.32. Lt7, chain length less than 7; gte7, chain length greater than or equal to 7.

A chain length could not be computed for 14 substances due to issues with resolving structures within RDKit. Only one of the 14 substances, DTXSID20153820, could be resolved since the chain length failed on account of the chain length exceeding the range used to calculate the maximum values. The chain length of this substance was manually annotated. The remaining 13 substances were dropped from further consideration. There were 31 substances that were tagged as “Not PFAS” based on the OECD structure definitions used within PFAS-Atlas. All were reassigned to the “unclassified” primary category. Fig. 2 is a bar chart showing the number of PFAS within each secondary category. The final PFAS landscape used for the remainder of the analysis comprised 15,525 substances.

Across the more than 15,000 PFAS substances evaluated, twenty-two percent (3430) of the substances fell into the “Aromatics PFAS, lt7” category. In addition, 1066 substances fell into the “Others, lt7” and 1180 in the “PFAA precursors, lt7” secondary categories. This represents a potential limitation of using broad definitions represented by the OECD primary categories themselves. The smallest secondary category was the “Others PFAS, cyclic, gte7” with 14 members whereas “PFAAs, cyclic, gte7” was a singleton.

A chemotype ToxPrint enrichment was explored following the approach outlined in Wang et al. [57] but using the PFAS specific ToxPrints developed in Richard et al. [44] (see Section S1 of the supplementary information for methodological details). This was an effort to identify whether there were specific structural features that might be helpful in splitting apart those primary categories with the largest memberships namely (i.e., “Aromatic PFASs”, “PFAA precursors” or “unclassified”). The most enriched features for the “unclassified” category included fluorotelomer chains and sulfonic acid functional groups whereas alcohols and carbonyls featured as functional groups for the “PFAA precursors”. No specific features were enriched for the “Aromatic PFAAs” categories. However, where there were enriched features, these were not determined to be sufficiently distinctive to justify creation of additional primary categories.

**Fig. 3.**
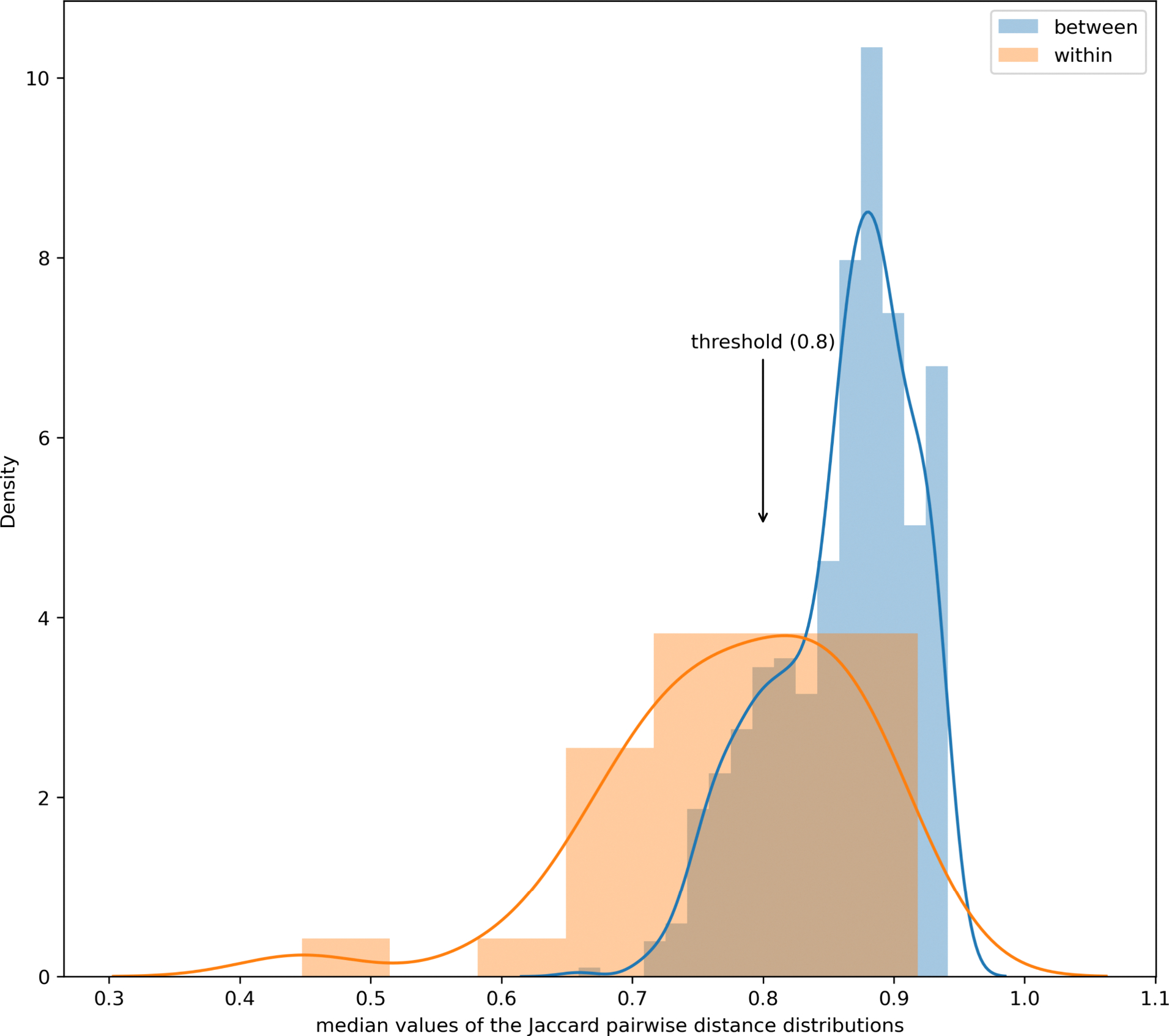
Probability density functions of the median Jaccard pairwise distance distributions for within (orange) and between (blue) secondary categories. Orange and blue graphed lines represent the fits to the probability density distributions. (For interpretation of the references to color in this figure legend, the reader is referred to the web version of this article.)

Structural similarity was evaluated within and between secondary categories to determine which secondary categories required further subcategorization (as discussed in Section 2.3.6 of the Methods). Fig. 3 shows the two distributions of the median pairwise distance distributions in the between and within secondary category combinations. The objective distance threshold derived by taking the 15^th^ percentile of the median pairwise distances from the between categories combinations resulted in a value of 0.8.

Based on the threshold, 16 secondary categories (Table 3) were found to exceed the value that would render them subject to further subcategorization. The sixteen secondary categories included the “Aromatic PFASs”, “PFAA precursors”, “Others” and “PolyFCA derivatives”. These categories are of little surprise given their membership sizes were the largest out of all the secondary combinations; hence, these categories were expected to be the most diverse in terms of their structural makeup. Since the “PFAAs, cyclic” category only comprised 1 substance, this was excluded from any subcategorization.

**Fig. 4.**
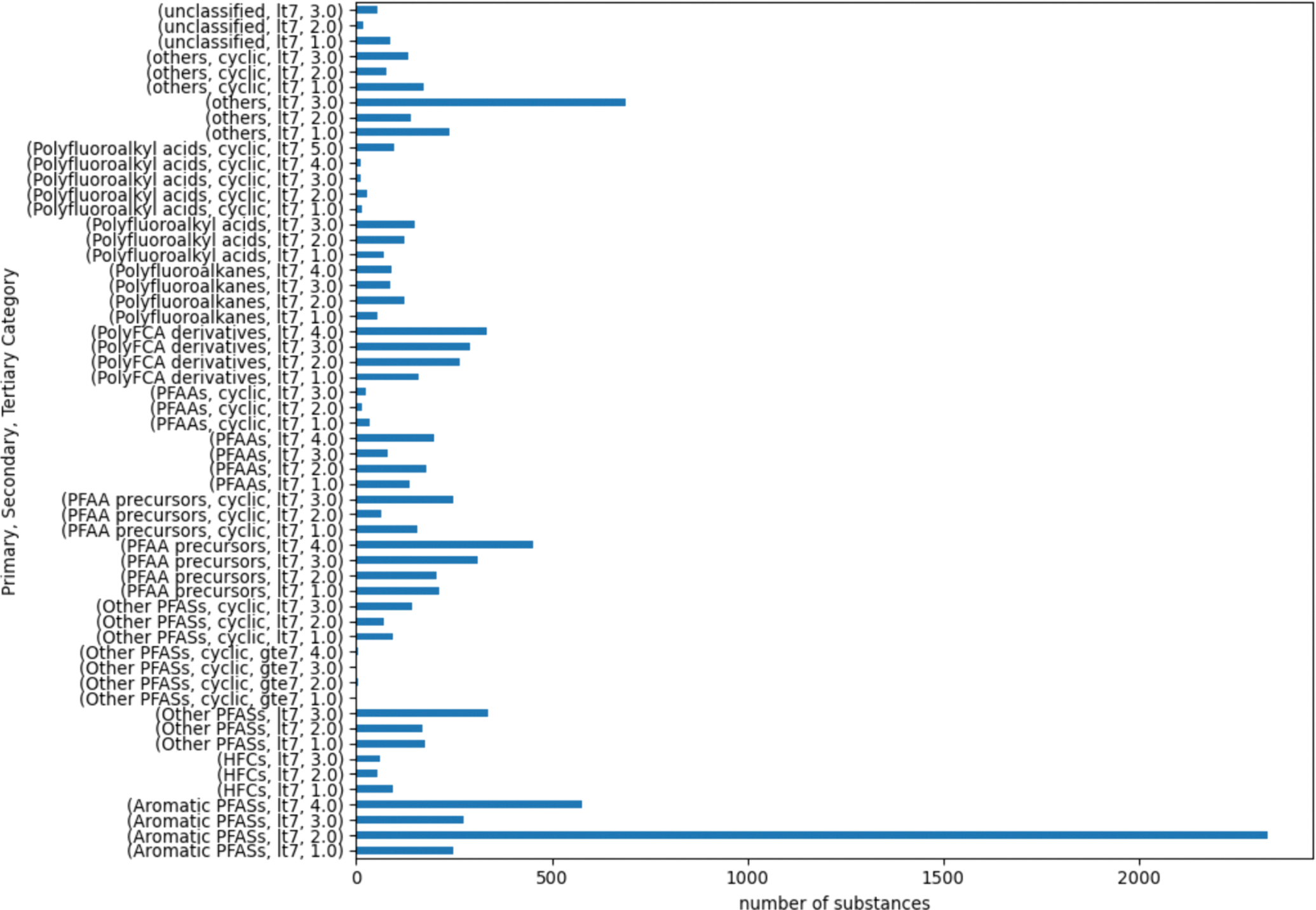
Bar chart showing the number of substances within each tertiary category, ordered by primary and secondary category roots. Methods to define tertiary categories are outlined in Section 2.7. Lt7, chain length less than 7; gte7, chain length greater than or equal to 7.

Fig. 4 shows the membership following the first generation of clusters being created for the 16 secondary categories that exceeded this objective threshold.

**Fig. 5.**
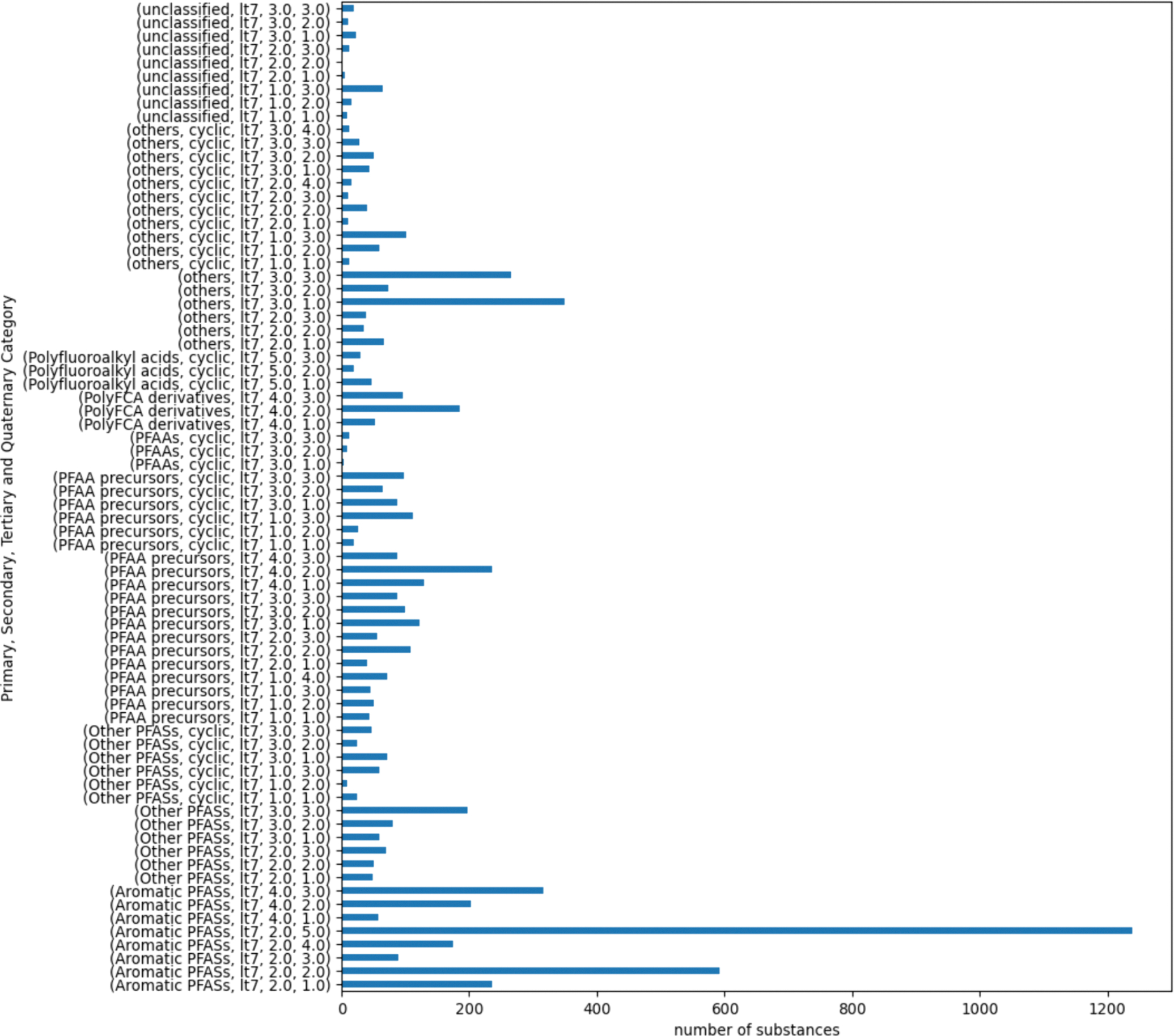
Bar chart showing the number of substances within each quaternary category, ordered by primary, secondary, and tertiary category roots. Methods to define quaternary categories are outlined in Section 2.7. Lt7, chain length less than 7; gte7, chain length greater than or equal to 7.

Following creation of the next generation categories, there were 23 tertiary categories that met or exceeded the threshold and were subcategorized further. The root primary categories were predominantly from the “Aromatic PFASs”, “Other PFASs”, and “PFAA precursors” categories. Fig. 5 reflects the quaternary categories for the 23 that were subset further.

Terminal categories were defined as either secondary or tertiary categories that did not exceed the threshold, as well as all quaternary categories. A total of 128 terminal categories (127 categories + 1 singleton (PFAAs, cyclic, gte7)) were ultimately derived. This represented a trade-off in terms of the final number of terminal categories that was a practical number to characterize the landscape of PFAS balanced with maximizing structural similarity within the categories themselves. The full list of 15,525 substances together with their terminal category assignments are provided as supplementary information. Structural similarity within categories did increase following subcategorization, Fig. S4 shows the ECDFs of several terminal categories which are left shifted relative to the original ECDFs for the secondary categories (Fig. S2), i.e. the pairwise distance range decreases.

### 4.2. Selection of representative substances

Whilst centroids were selected as the most representative substance from each terminal category, there was a recognition that a single chemical was unlikely to capture the breadth of diversity within a category. Additional substances to capture the breadth and structural diversity relied on the MaxMinPicker method [50]. This method was used to select up to 3 further substances in addition to the centroid. A total of 484 substances were selected using this approach for 121 of the terminal categories. Terminal categories with 5 or fewer members did not result in any additional substances being selected (beyond the centroid) by the approach. Table 4 lists the 7 terminal categories which had insufficient membership to apply the MaxMinPicker approach.

To evaluate the proportion of structural diversity captured by the selected representative substances, the normalized cumulative minimum distance was calculated as a function of the number of substances selected using the MaxMinPicker method as discussed in Section 2.3.9. There were 15 terminal categories, out of the 121 terminal categories for which diverse substances were selected, where picking 3 substances captured at least 50% of the structural diversity (shown in Table 5).

For the largest terminal category, “Aromatic PFASs, lt7, 2.0, 5.0”, selecting up to 3 diverse substances only captured 0.8% of the structural diversity. In order to capture 80% of the structural diversity for this terminal category, 528 substances would need to be selected for data collection. The number of substances that needed to be selected from each terminal category to capture 80% of the structural diversity varied from 3 (as shown above in Table 5) to 528 with the median number being 30.

**Fig. 6.**
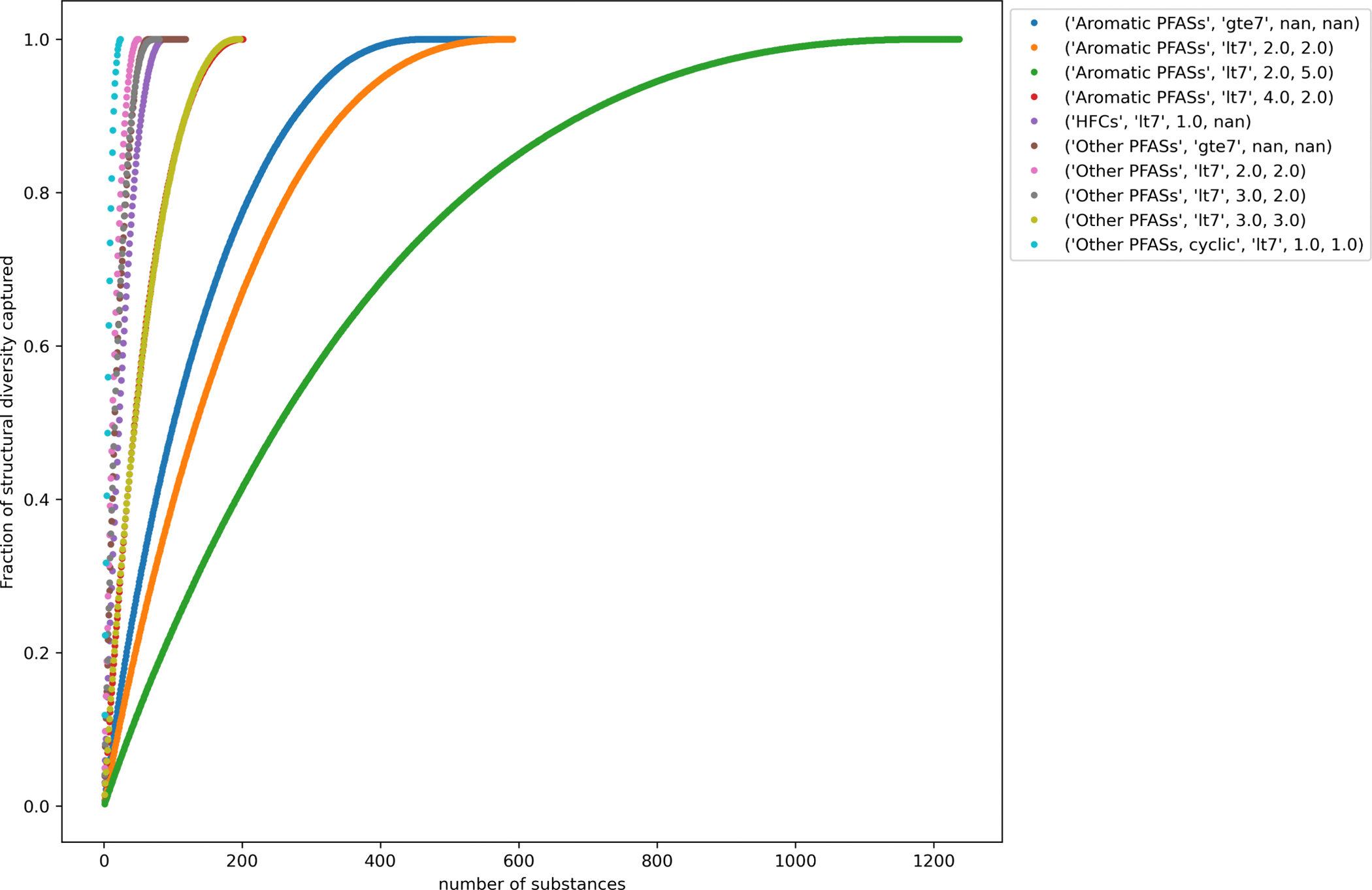
For a selection of terminal categories, the extent to which the fraction of structural diversity is captured relative to number of diverse chemicals selected varies.

**Fig. 7.**
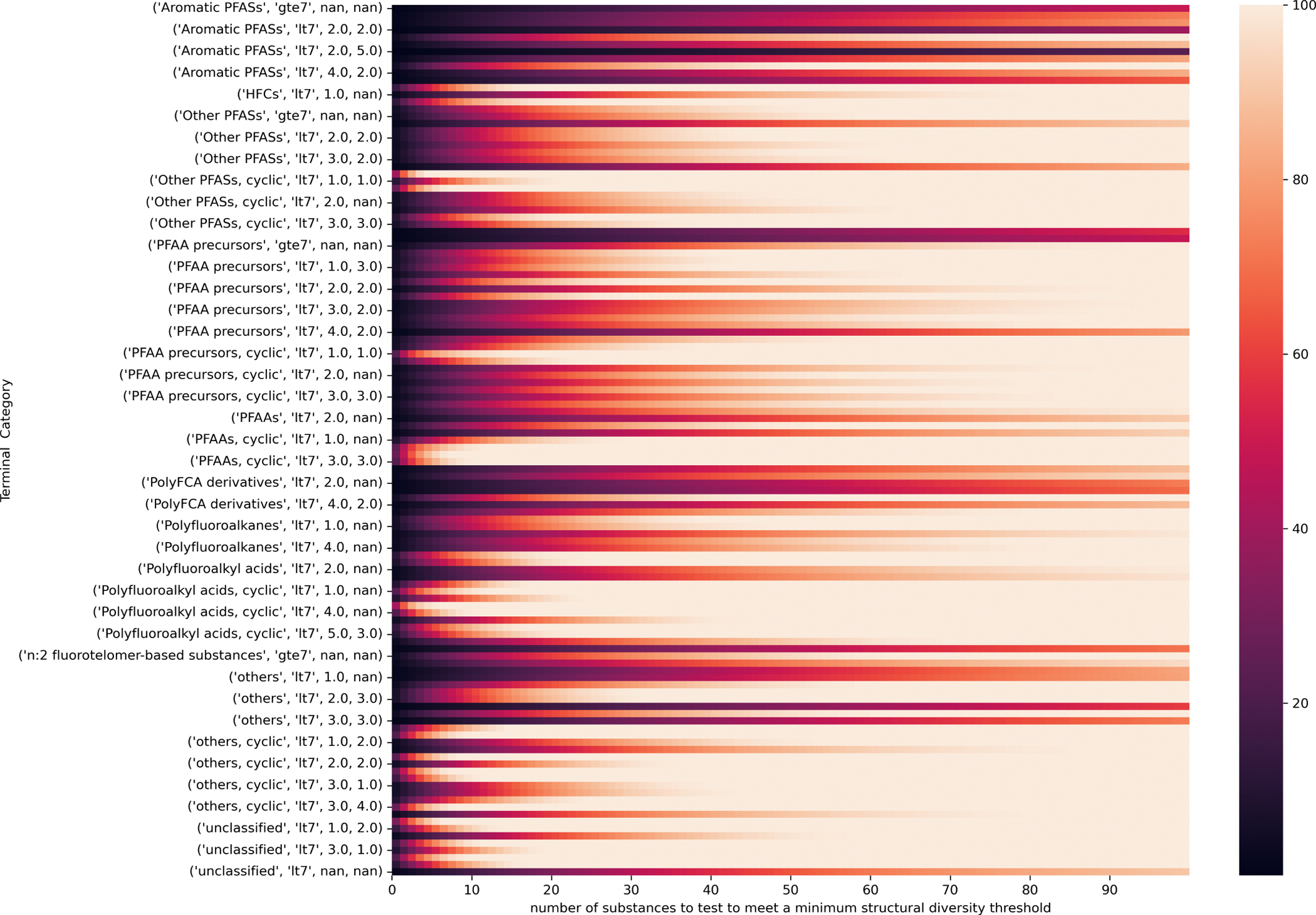
Heatmap showing the number of diverse substances that would need to be selected to achieve a specific minimum % structural diversity coverage across each terminal category. A selection of terminal categories are plotted to highlight how the number of diverse substances needed varies. The legend color corresponds to the number of substances needed as presented on the x axis.

Fig. 6 shows the curves of the number of diverse selections as a function of the percentage normalized cumulative minimum distances for 10 representative terminal categories. These vary in steepness showing how quickly or not the structural diversity coverage converges with number of diverse selections depending on the terminal category of interest. Fig. 7 highlights the difference in the number of diverse chemicals that would be needed to capture a minimum structural diversity across each terminal category.

**Fig. 8.**
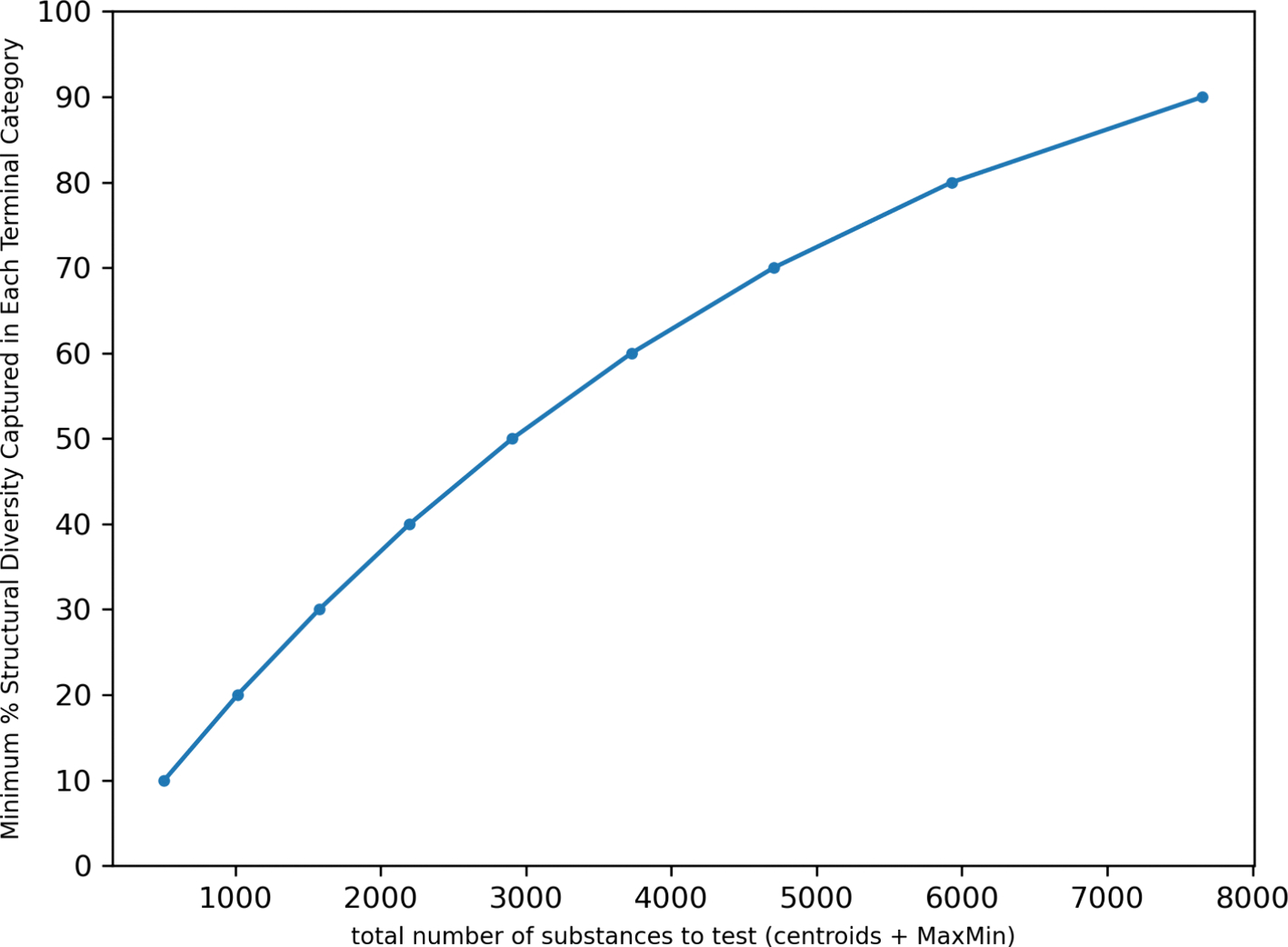
Lineplot showing the number of diverse substances that would need to be selected to achieve a specific minimum % structural diversity coverage across all the terminal categories. To achieve 80% structural diversity, a total of 5929 substances would need to be selected across the various terminal categories.

Fig. 8 attempts to summarize the tradeoff of the number of diverse chemicals (thus the centroids and MaxMin) as a function of % structural diversity captured across the terminal categories.

The diverse selections identified earlier for the terminal categories reflects a pragmatism in terms of identifying a potential candidate list of substances. As discussed later in Section 4.6, the structural diversity captured forms one of the considerations in selecting candidates for additional data collection relative to those terminal categories that are data poor or contain substances that are on the TSCA inventory.

### 4.3. Evaluation of physical state and physicochemical consistency within terminal categories

In order to determine the nature of further data collection activities and understanding the potential presence in different environmental media, physical state and physicochemical information was determined for the PFAS landscape as far as possible. For the 15,525 substances in the PFAS landscape, 431 substances (2.8%) could not be assigned into any specific physical state and physicochemical designation owing to a lack of predicted physicochemical property information. The designations of the remaining substances are shown in Table 6.

**Fig. 9.**
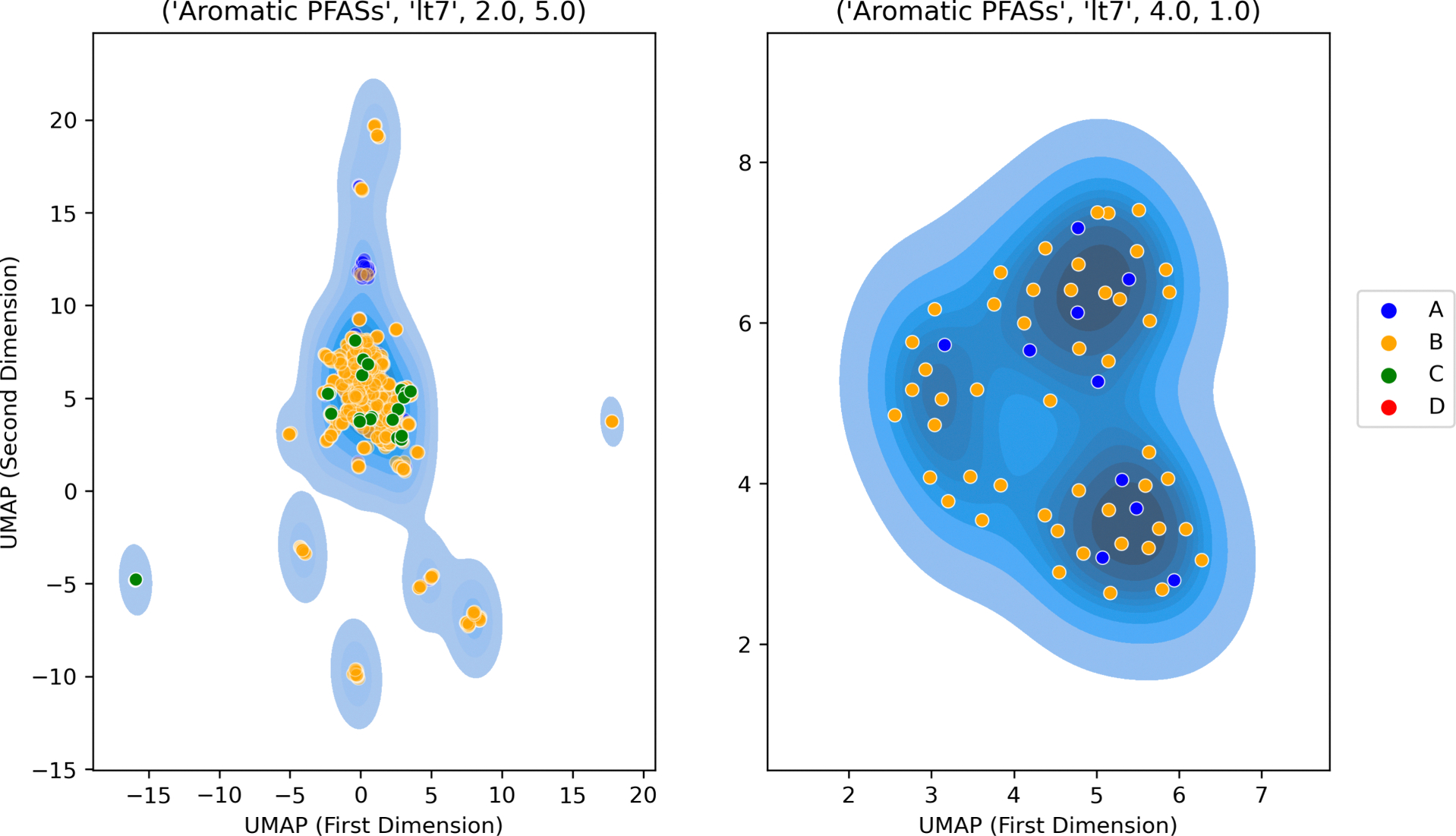
UMAP projections for terminal category a) “Aromatic PFASs, lt7, 2.0, 5.0” and b) “Aromatic PFASs, lt7, 4.0, 1.0” using Morgan chemical fingerprints with physical state and physicochemical designations A-D overlaid.

The high percentage of substances in designation C additionally raises questions about the compatibility with most NAM-based systems. Across the terminal categories, there was a general trend of number of different designations increasing with size in category membership (see Fig. S5) Fig. 9 shows an example of one of the most diverse and largest terminal categories “Aromatic PFASs, lt7, 2.0, 5.0” which comprises 1238 members and spans 3 of the 4 designations. Although substances predominantly lie within designation B, there is no discernible separation between the designations across the structural category as characterized by Morgan fingerprints. In contrast all 96 substances belonging to terminal category “PolyFCA derivatives, lt7, 4.0, 3.0” fell into designation B (figure not shown) whereas the 58 substances in “Aromatic PFASs, lt7, 4.0, 1.0” fell into designations A and B. There was a positive association between how structurally similar a terminal category was and the consistency in physical state and physicochemical profile observed (as reflected by the designations). However, the Morgan fingerprints could not resolve all the differences. For the selection of potential candidates for data collection, the physical state and physicochemical profile remains an important consideration in concert with the structural diversity described in Section 4.2.

### 4.4. Variation of POD values across and within terminal categories

Ultimately, the terminal categories are intended to facilitate a read-across for human health assessment. To explore the feasibility of this further, the 25^th^ percentile values of oral BMDhs were calculated using available non-cancer data for 55 substances and repro/developmental toxicity data for 35 substances. The distributions were plotted in a series of box plots. *In vivo* toxicity data were available for at least one chemical in 28 of the 128 terminal categories across the two study types (28 for non-cancer, 19 for repro/developmental). The available data allowed preliminary trends for terminal categories to be observed where the primary root was Aromatic PFASs, PASF-based substances, PFAAs, Polyfluoroalkanes and Polyfluoroalkyl acids categories (see Fig. S6 in the Supplementary Information for the boxplots for both study types).

**Fig. 10.**
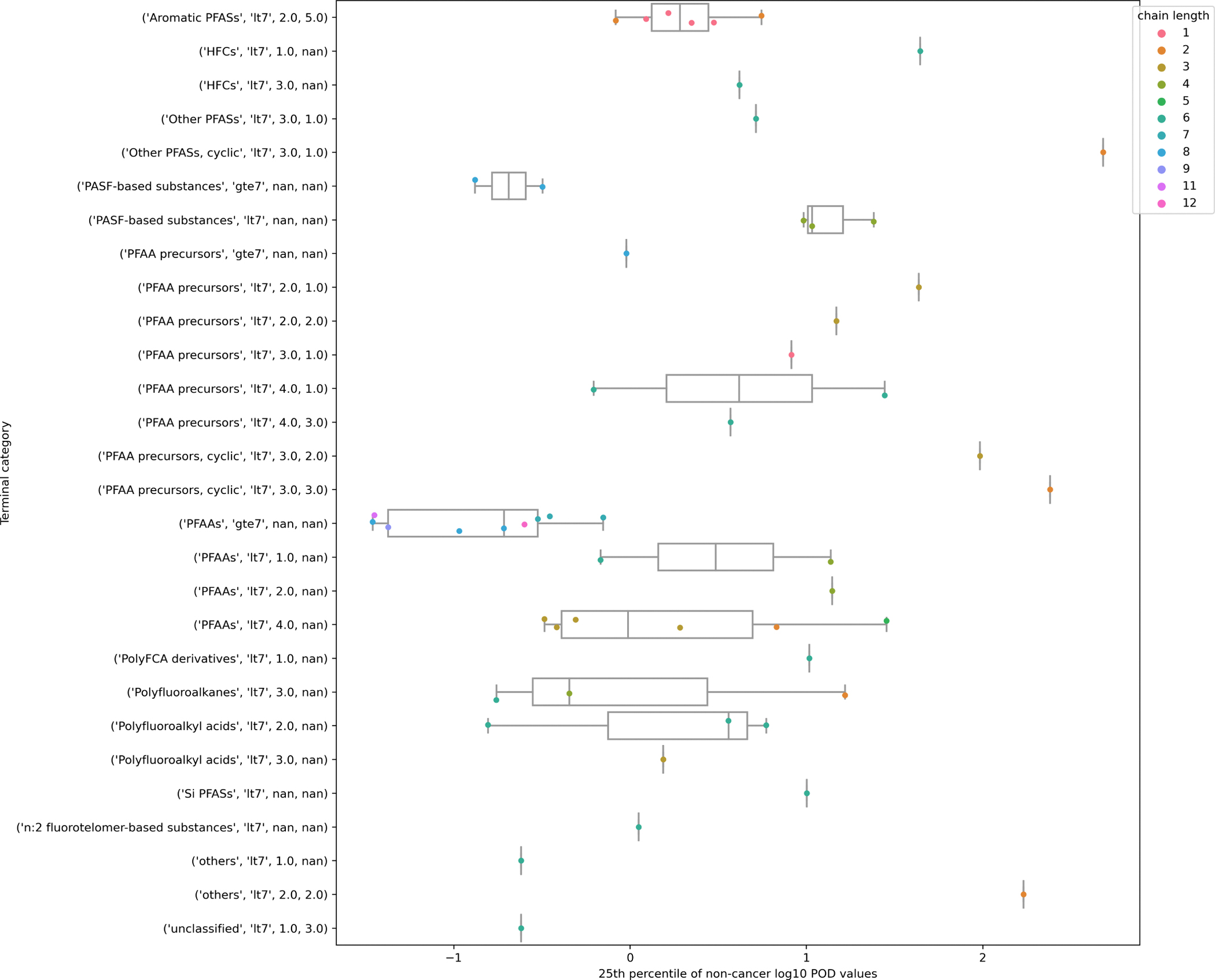
Boxplots showing the spread of the 25^th^ percentile of the oral non-cancer log 10 POD values across and within terminal categories bounded by the carbon chain number. The box in the boxplot reflects the quartiles of the dataset, whilst the whiskers extend to + 1.5 * inter-quartile range (IQR). Outliers are shown as points if they exceed 1.5 * IQR.

Fig. 10 shows boxplot and strip plots for the oral non-cancer studies only. It appears that substances at each end of the spectrum of chain length within a category tended to exhibit lower toxicity, i.e., their aggregate POD is higher. The spread of POD values within a category with greater diversity in chain length tend to span ~1–2 orders of magnitude.

Although the available toxicity data are limited, there does appear to be some separation in the potency distributions between terminal categories based on a common primary root. Inspection of Fig. 10 does show a shift in potency values between the PFAA categories with a left shift for those substances in the gte7 category vs the majority of the PFAA lt7 categories. A similar shift was observed for the PASF-based categories. However, the relatively large spread for some of the terminal categories suggests that additional refinement beyond structural similarity and chain length (such as factoring in toxicokinetic information) will likely be needed for some terminal categories prior to broader application in a read-across context.

### 4.5. Qualitative mechanistic and toxicokinetic designations

**Fig. 11.**
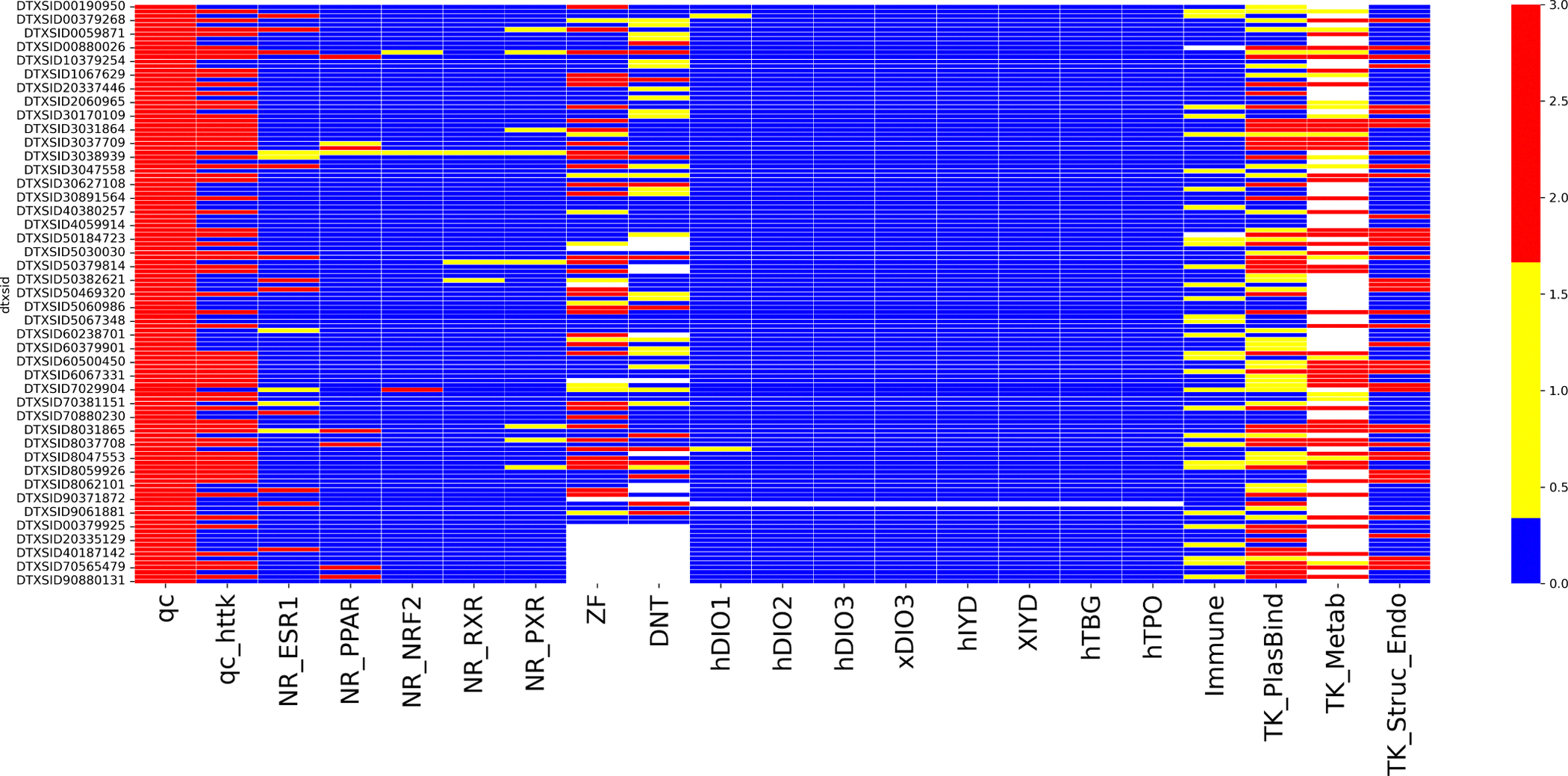
Heatmap of NAMs flags for the ~ 150 PFAS substances (~120 of which passed analytical QC (qc)) tested as part of the research programme described in Patlewicz et al. [23]. Y axis tick labels do not capture all substances, only every 3rd substance by DTXSID is shown. qc = analytic QC and qc-httk analytical QC for TK; ESR1 = Estrogen Receptor 1; PPAR=peroxisome proliferator- activated receptor; NRF2 = nuclear factor erythroid 2-related factor 2; RXR=retinoid X receptor; PXR=pregnane X receptor; ZF=zebrafish; DNT=developmental neurotoxicity; DIO1, DIO2, DIO3 = Type 1,2,3 deiodinase; IYD=iodotyrosine deiodinase; TBG=thyroxine binding globulin; TPO=thyroid peroxidase. No data were represented as null values (white colored), data available but no flag identified as denoted a 0 (blue colored), 1 denoted a medium confidence flag (yellow colored) and 2 was associated with a high confidence flag (colored in red) consistent with the descriptions described in Table 2. (For interpretation of the references to color in this figure legend, the reader is referred to the web version of this article.)

There were six data streams with qualitative flags assigned for the ~ 150 PFAS tested as part of the research project described in Patlewicz et al. [23] namely: 1) nuclear receptor assays (NR); 2) developmental toxicity (zebrafish testing); 3) DNT (developmental neurotoxicity); 4) thyroid toxicity; 5) immunosuppression (BioMAP assays); and 6) toxicokinetics (TK). Fig. 11 profiles all the NAM flags across the different technologies together with a stock QC flag [30] (Pass (red)) and a qc_httk flag (Pass (red)).

From Fig. 11, the first two columns represent the quality control (QC) information. The next 5 columns represent the NR data. The next 2 columns represent the developmental toxicity (ZF) assay and the DNT assay. The next 8 columns represent the thyroid assay outcomes followed by the integrated immunotoxicity flag from the BioMap assays. The last 3 columns represent the TK flags.

A semi quantitative analysis was performed by computing which PFAS ToxPrints were enriched for each NAM flag (excluding the TK_Metab and TK_Struc_Endo flags since this information was captured using the predictions from the Dawson et al. [56] model). The full set of enriched ToxPrints are provided in the supplementary information. Those enriched PFAS ToxPrints were then used to profile the entire landscape to provide a predicted NAM flag profile. Performance metrics were derived to compare the actual and predicted NAM flag (see Table 7). Performance appeared weakest for the DNT and Immune flags which had the fewest number of ToxPrints that were enriched. It is worth noting that only ~ 150 chemicals (of which 124 substances overlapped with the PFAS landscape) were tested and the performance of these ToxPrint signatures may change as more substances are tested.

Predictions of half-life in humans were made for all the 15,525 substances. 69% of the substances were predicted to have the slowest half-life of greater than 2 months (bin 4), 14% in the next slowest bin (bin 3, 1 week – 2 months) and the remaining 17% in the second fastest bin (bin 2, 12 h – 1 week).

**Fig. 12.**
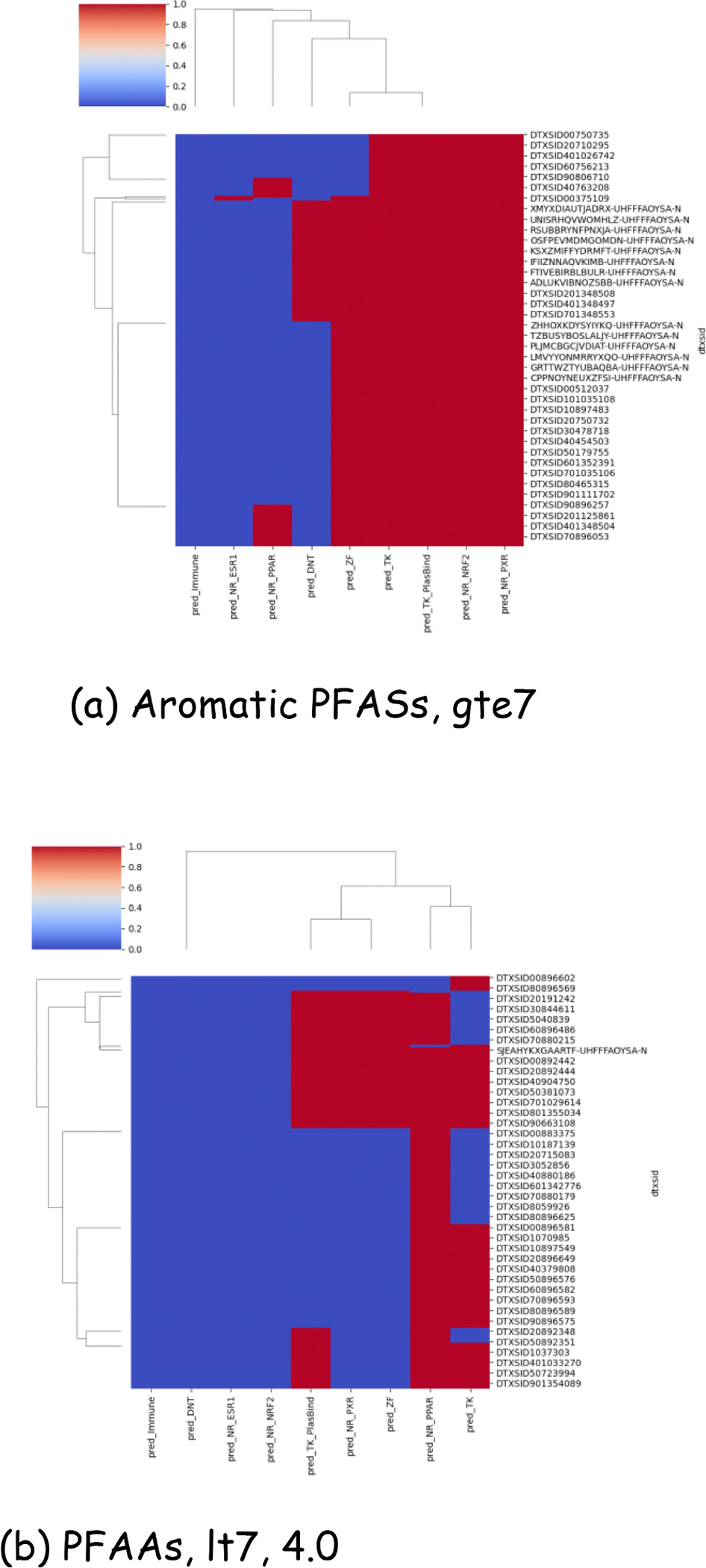
Clustermaps for terminal category “Aromatic PFASs, gte7” and “PFAAs, lt7, 4.0” to illustrate their concordance across predicted NAM profiles.

To demonstrate the integration of the mechanistic and TK-related data with the structural categories, two terminal categories were clustered based on the predicted mechanistic and TK enrichment flags as shown in Fig. 12. Terminal categories “Aromatic PFASs, gte7” and “PFAAs, lt7, 4.0” were first profiled against the enriched PFAS ToxPrints in conjunction with the predicted half-lives and then clustered to show the potential NAM profile across the entire category and how consistent it was across the terminal category members. Terminal category “Aromatic PFASs, gte7” had a far more consistent profile whereas “PFAAs, lt7, 4.0” showed some differences in the half-life predictions which might warrant further additional substances to be identified for data collection to reflect the TK diversity.

### 4.6. Potential application to support the National PFAS testing Strategy (NTS)

There are several considerations that come into play when identifying potential candidates for data collection in concert with the landscape defined. To make the NTS actionable, one consideration was to limit the landscape to one that was constrained by the TSCA active inventory to increase the feasibility of being able to identify a manufacturer/importer of the substance. A second enables the tradeoff between the number of diverse substances to select vs capturing the structural diversity to be more practically addressed. Herein, the scope of terminal categories represented by the full TSCA and TSCA active inventory and the impact this had in terms of capturing structural diversity was evaluated. Finally, a proposal was outlined that considers how the terminal categories could be triaged to initially focus on terminal categories which were either data poor or contained members that represented large exposure sources.

#### 4.6.1. Constraining the landscape to the TSCA active inventory

##### 4.6.1.1. TSCA inventory.

**Fig. 13.**
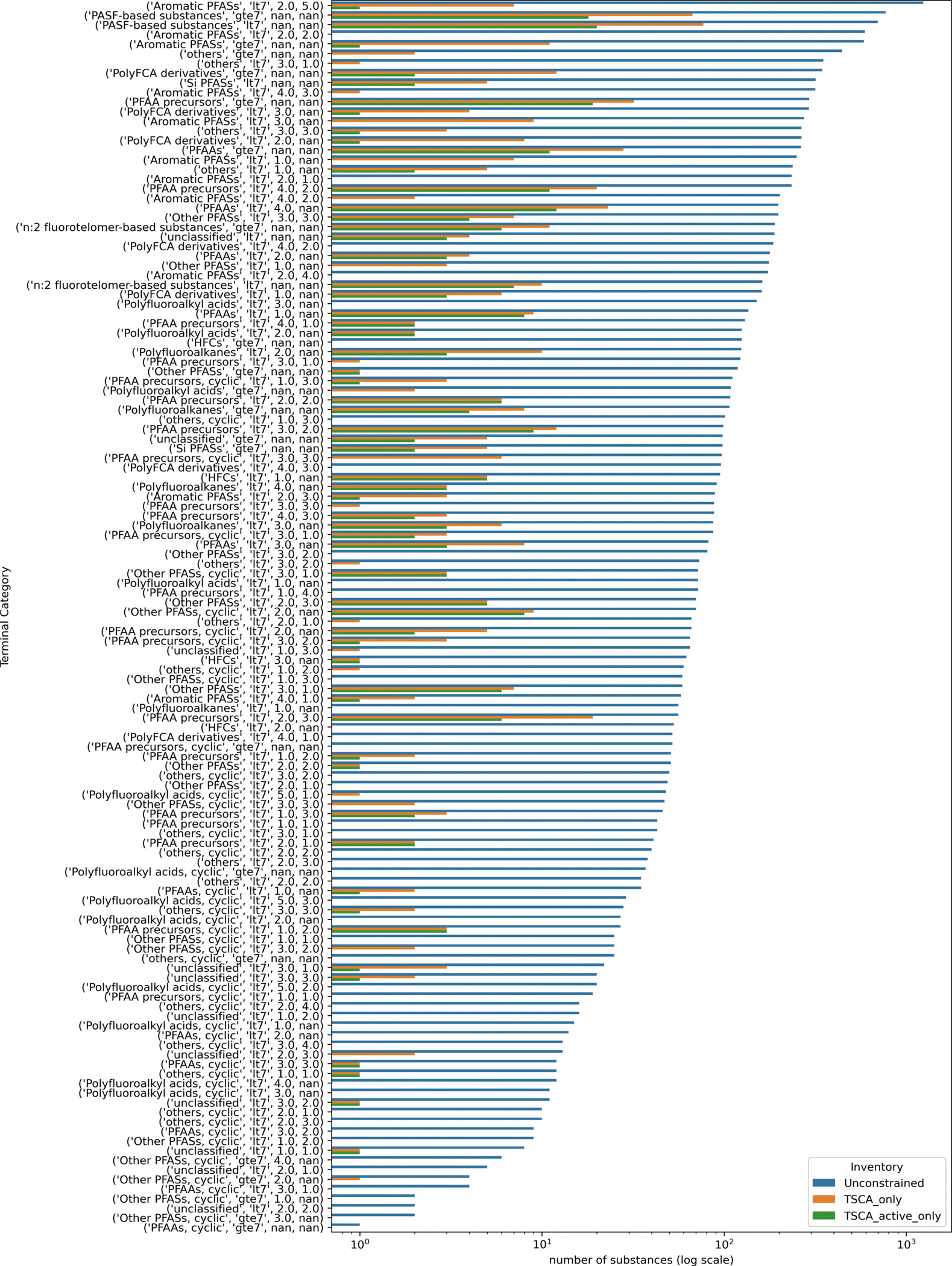
Bar chart showing membership of terminal categories and how that differs when constrained by TSCA inventory or TSCA active inventory.

Of the substances in the PFAS landscape, only 563 substances were identified to be on the TSCA inventory, of which 237 were ‘active’ and the remaining 326 ‘inactive’. Active and inactive refers to the EPA’s designation of whether a substance is active in US commerce based on the rule requiring industry to report chemicals manufactured or imported or processed in the US over a 10 year period ending 21st June 2016. There were 384 substances in the full landscape that matched a degradant of a TSCA substance. Fig. 13 shows a bar chart of the membership of the terminal categories and how that differs when considering TSCA inventory status (overall or by active TSCA only).

The largest category memberships when constrained by presence on the TSCA inventory reflected the “PASF-based substances, lt7”, “PASF-based substances, lt7” and “PFAA precursors, gte7” categories. Across the terminal categories, 63% of the categories (80 out of the 128 categories) contain members on the TSCA inventory. If only categories containing substances that are on the TSCA active inventory are considered, then the number of terminal categories decreases to 60, i.e., 47% coverage. Some of the categories where there were no examples on the TSCA inventory were fairly large in size, examples include several of the Aromatic PFASs categories with 174–592 members as well as the PolyFCA derivatives and Polyfluoroalkyl acids with 186 and 151 members respectively.

##### 4.6.1.2. Selection of representative substances in the constrained TSCA active inventory.

**Fig. 14.**
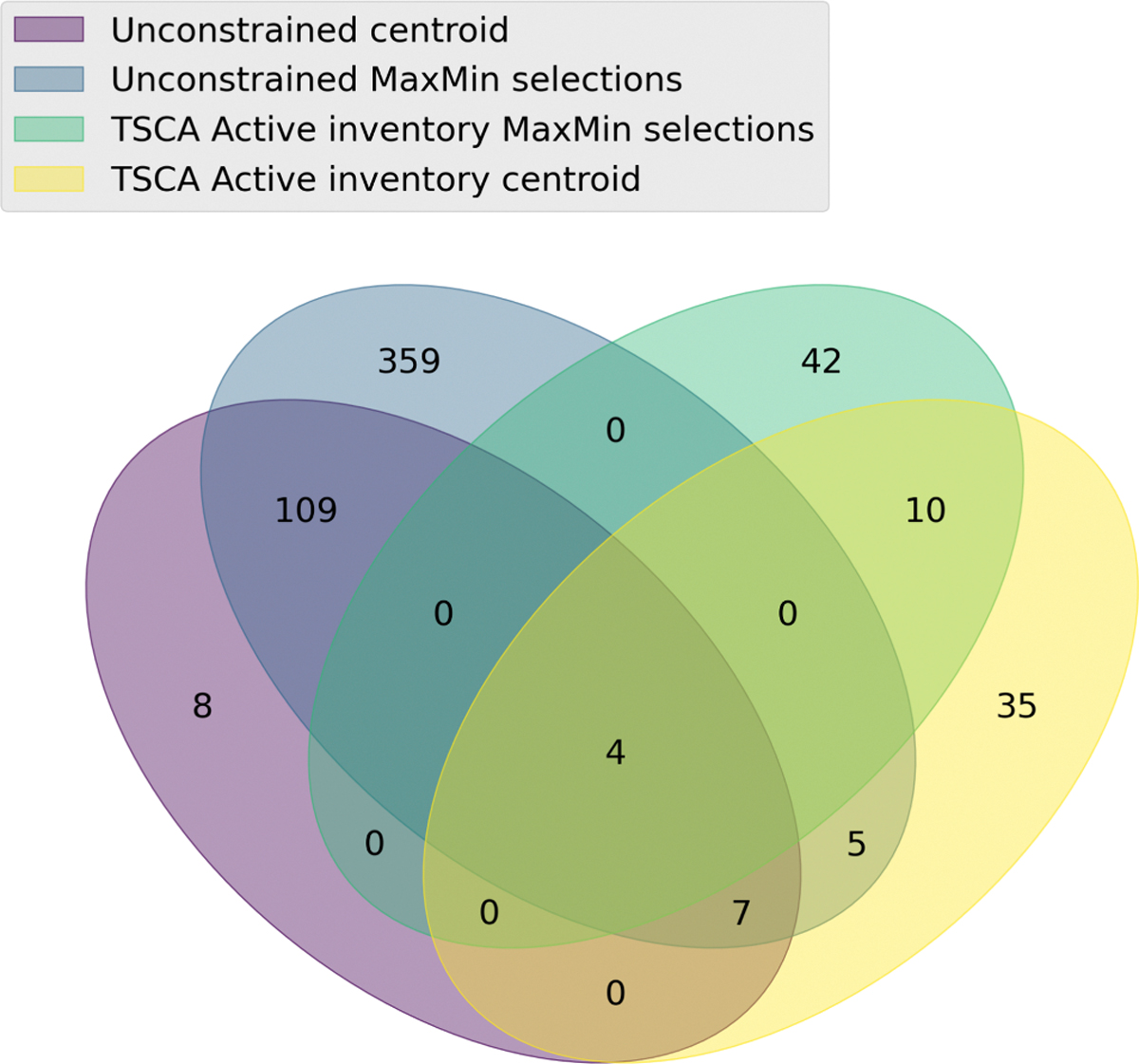
Venn diagram showing the overlap in substances based on whether they were identified as additional diverse picks or centroids in the unconstrained PFAS landscape and that constrained by the TSCA active inventory.

Centroids were computed for the 60 terminal categories containing substances that were on the active TSCA inventory. For 14 of these terminal categories, membership exceeded 5, which permitted the MaxMinPicker approach to be applied to identified further analogues. An additional 56 analogues were selected from this constrained landscape. Fig. 14 shows the overlap in substances (centroids and diverse) across the unconstrained and the TSCA active constrained landscapes. The minimal overlap between the sets highlights the limitations of using a constrained landscape, i.e., one which does not represent the breadth of the PFAS chemistry. However, the substances on the TSCA active inventory represent those substances that are currently in commerce in the US and potentially represent the largest exposure source. It is worth noting that the overlap in the venn diagram in terms of exact substances may not reflect the overlap in structurally similar substances.

An evaluation of the structural diversity captured using the centroids and additional MaxMin substances relative to the number of substances that would need to be selected to attain 80% structural diversity coverage was also undertaken in the same manner as had been performed for the full landscape. For the 13 of the 14 categories where the MaxMin approach had been applied, the diverse picks originally selected captured more than 50% of the structural diversity as shown in Table 8. This is not so surprising given the TSCA active set substantially limited the terminal category size and in turn their diversity.

**Fig. 15.**
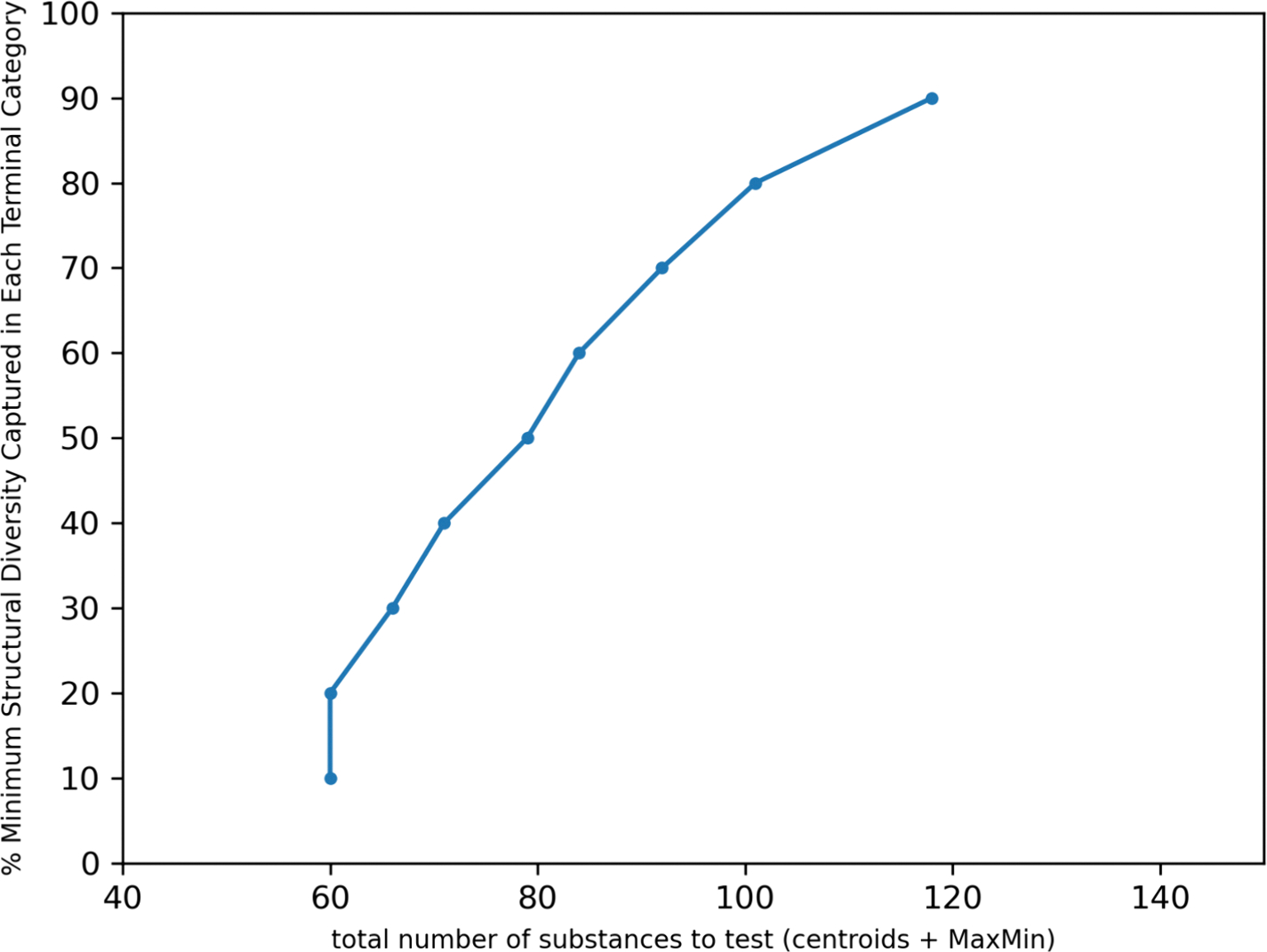
Lineplot of the TSCA active constrained terminal categories as a function of number of diverse substances selected and the minimum % structural diversity captured across all terminal categories.

For the largest terminal category, “PASF-based substances, lt7”, 3 diverse substance selections captured 47.72% of the structural diversity. In order to capture 80% of the structural diversity for this terminal category, 7 substances would need to be selected for additional data collection. The number of substances to select from each terminal category to capture 80% of the structural diversity varied from 1 to 7 with the median number being 4. Across the entire TSCA active space, considering the 14 categories where a MaxMin approach could be applied – 55 substances would need to be selected capture a 80% structural diversity. In order to capture a minimum of 80% structural diversity across all the TSCA active categories, at least 101 substances (the centroids for categories where no MaxMin had been applied + MaxMin) would be ideally selected for data collection. Fig. 15 summarizes the structural diversity attained across all TSCA active terminal categories.

#### 4.6.2. Proof of concept Workflow: Identifying potential candidates for data collection

**Fig. 16.**
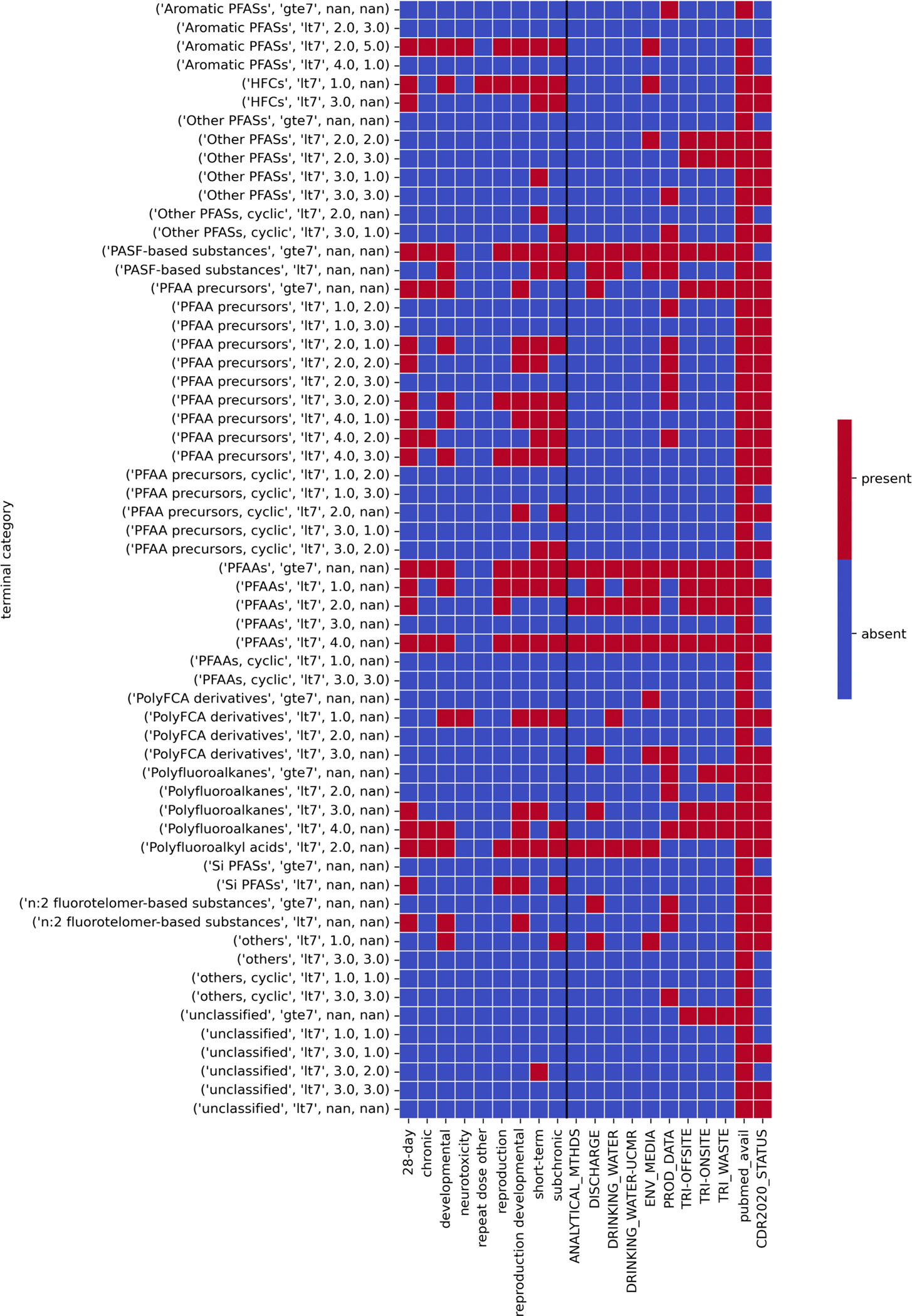
Heatmap of toxicity data availability and qualitative exposure and release designations. Notes: pubmed_avail is a tag to denote presence or absence of articles indexed in Pubmed. PROD-Data = Production data, DISCHARGE=Discharge Monitoring data, DRINKING_WATER=Drinking Water (State) Data, DRINKING_WATER-UCMR=Drinking Water data comprising Unregulated Contaminant Monitoring Rule data and State level monitoring data, ENV_MEDIA=Environmental Media data, TRI_Waste = Toxics Release Inventory (TRI) Data Waste Managed, TRI_On-Site = On Site TRI Data, TRI_Off-Site = Off Site TRI Data, Analytical_Mthds = PFAS with Validated Analytical Methods 533 and 537.

The availability of toxicity data across different study types and the presence of substances within different monitoring lists was arrayed across the terminal categories. All oral and inhalation studies from ToxValDB 9.5 were first retrieved. There were 76 substances with data for one or more of the study types which were then matched on the basis of DTXSID with substances in the PFAS landscape. The resulting table was then transformed to produce a table where columns represented different study types, rows were substances and cells were labelled 1 if data for a specific study type existed for a specific substance and 0 if no data existed. The qualitative lists reflecting various priorities, environmental detection/discharged, and availability of analytical methods etc. as described in Section 2.4.1 were compiled together and transformed into a table where rows represented substances, columns represented the different list sources and cells were populated with a 1 or 0 to denote presence or absence on a specific list. There were 448 substances identified across these lists but only 198 unique substances which were then matched with substances in the PFAS landscape. CDR status tags and Pubmed count tags were then added to the PFAS landscape. Columns representing the toxicity study types and various lists were grouped by terminal category to produce a new table which reflected presence or absence of information (denoted by 1 or 0). Study quality was not considered – only the availability of publicly available toxicity data. The set of terminal categories were filtered to retain only those terminal categories which contained members on the TSCA active inventory (60 terminal categories). Fig. 16 provides a perspective of this information, namely the toxicity data sparsity across the categories that fall within the scope of the TSCA active inventory as well as different environmental monitoring efforts or discussed in the literature. The PFAAs categories and their subcategorizations show up with data entries which is largely unsurprising, given the extent to which PFOA and PFOS have been studied. Note: The figure provides a landscape perspective of the data coverage across broad structural categories which may not entirely align with toxicological classes.

**Fig. 17.**
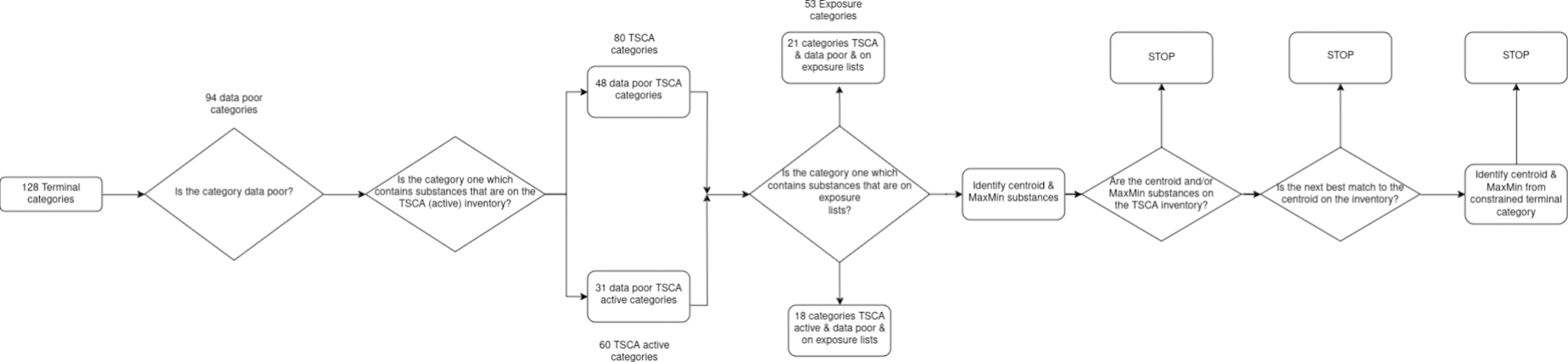
Workflow to highlight the main steps involved in prioritizing potential candidate selection for data collection for a given terminal category.

Each of the earlier sections in of themselves highlight different lines of evidence that can inform the identification of potential candidates for data collection. Note: The qualitative release designations are not intended to be exhaustive but could be refined to factor other relevant information data streams such as existing epidemiological studies etc. Here, an attempt was made to demonstrate how these steps can be integrated together to triage terminal categories and their potential candidates for subsequent tiered data collection efforts (Fig. 17). Step 1 is to consider a given terminal category and determine whether it meets the condition of being a ‘data poor category’. Data-poor in this context was to consider whether this was a category that did not contain any members for which repeated dose toxicity data existed (by the oral or inhalation route and with a reported NOAEL, LOAEL, LOEL, NOEL, NEL or LEL value). Note in this study, only repeated dose toxicity data within the publicly available ToxVal was considered. There were 94 terminal categories out of the 128 total number of categories that met this condition.

The next step was to focus on terminal categories that overlapped with those which contained substances that were on the TSCA inventory.

There were 80 terminal categories that contained substances that were on the TSCA inventory of which 60 terminal categories contained substances that were on the TSCA active inventory. Of the TSCA categories, 48 also satisfied the condition of being a ‘data poor’ category. In contrast, 31 of the TSCA active categories were ‘data poor’. The following step was to consider terminal categories that contained substances that were on different environmental monitoring (EM) lists. There were 53 terminal categories that contained substances that were on one or more monitoring lists or 117 if Pubmed article availability was taken into account. Of these EM terminal categories, 21 were also overlapping with data poor TSCA categories or 18 of the data poor TSCA active categories. The 18 terminal categories included “Aromatic PFASs, gte7”, “Other PFASs, lt7, 2.0, 2.0”, “PFAA precursors, lt7, 1.0, 2.0”, “PolyFCA derivatives, gte7”, “Polyfluoroalkanes, gte7” and “unclassified, lt7, 2.0, 1.0”. Note: Only 2 of these categories met the condition to apply the MaxMin approach – taking into account those categories for which 3 substances would need to be selected to achieve 80% structural diversity, the remaining 16 categories would be limited to selecting the centroid.

For a category that satisfied all these conditions, the next step would be identify the representative substances characterizing the category (namely the centroid and MaxMin substances and check whether any were on the TSCA inventory). If none of these were on the inventory, then the next step would be to check whether the next closest match to the centroid was on the inventory. If not, the next steps would be to identify the centroid and MaxMin substances from either the TSCA constrained landscape or the TSCA active constrained landscape for that terminal category. Fig. 17 summarizes these steps in a conceptual workflow.

**Fig. 18.**
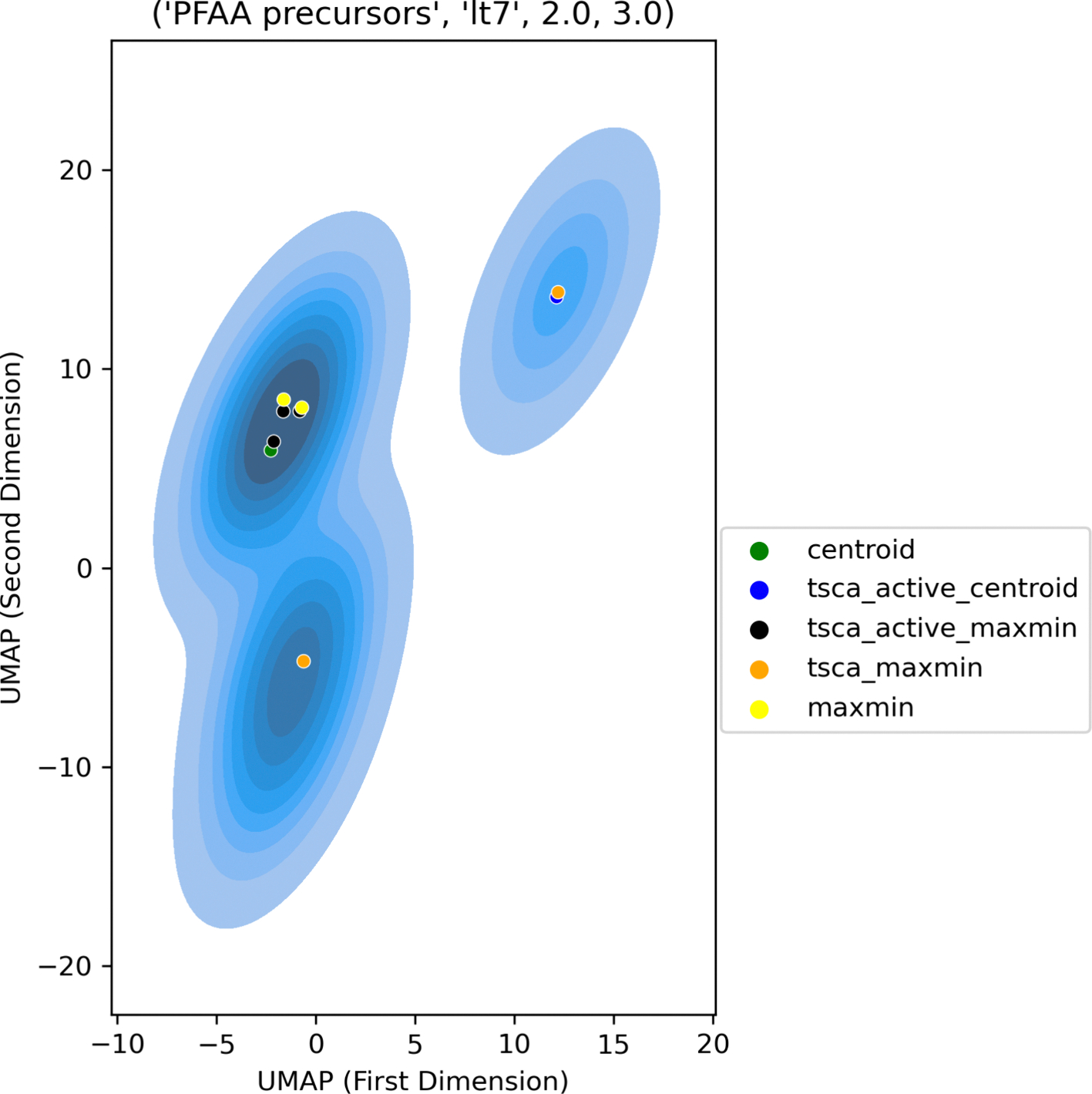
UMAP projection of terminal category “PFAA precursors, lt7, 2.0, 3.0” with its (TSCA active) centroid and MaxMin substances shown.

For illustrative purposes, terminal category “PFAA precursors, lt7, 2.0, 3.0” was identified that met the conditions of being a data poor category, containing members on the TSCA active inventory and containing members on various environmental monitoring lists, discharge and TRI lists. This terminal category comprises 56 members. If the category were constrained by TSCA active substances only, the category size would be reduced to 6 members of which 2 substances would capture 80 % of its structural diversity. The centroid, DTXSID70884511 was on the TSCA inventory but the TSCA active centroid DTXSID60880406 could be selected. Fig. 18 shows a UMAP projection [36] with the centroid, MaxMin and TSCA centroid substances shown for illustrative purposes to highlight their relative positions in the structural space captured within the terminal category.

### 4.7. Operationalizing the terminal categories for re-use

There were 11 terminal categories that had memberships of less than 10 which were aggregated into one miscellaneous group. A random forest classifier was trained to assignment membership of a substance into one of 118 categories (117 terminal categories + a miscellaneous category). The features used were Morgan fingerprints, chain length and primary category assignments whereas the labels were the terminal category names. A random forest classifier with default settings applied as part of a 5-fold stratified CV procedure gave rise to a mean balanced accuracy of 0.808 (std 0.014). A randomized search CV procedure using a range of different hyperparameters found the highest balanced accuracy (BA) to be with 400 trees, a minimum split size of 2, a minimum number of data points allowed in a leaf node to be 2, a maximum features to be the square root of the number of features, and a balanced subsample class weight. The CV mean balanced accuracy was determined to be 0.845 (std 0.013). This model was then applied to the test set that had been held out. The balanced accuracy of the test set was determined to be 0.857. The BA varied across the terminal categories with a median value of 0.973 and a minimum value of 0.50. The worse performing categories were the unclassified categories namely “unclassified, lt7, 3.0, 1.0”, “unclassified, lt7, 3.0, 2.0”, “unclassified, lt7, 3.0, 3.0” and “unclassified, lt7, 1.0, 3.0” all of which had BA less than or equal to 0.5. There were 8 categories with a BA less than 0.75 (of these 4 had a BA less than or equal to 0.5). The top 5 highest performing categories were “Aromatic PFASs, lt7, 2.0, 3.0”, “others, cyclic, lt7, 3.0, 3.0”, “others, cyclic, lt7, 1.0, 1.0”, “PFAA precursors, cyclic, lt7, 1.0, 1.0” and “PFAAs, cyclic, lt7, 2.0”. The full test set performance scores and the feature importances are provided in the supplementary information. Fig. S8 shows the BA and recall scores across the terminal categories.

## 5. Conclusions

EPA was directed by Congress to develop a process for prioritizing which PFAS or classes of PFAS should be subject to additional research efforts based on potential for human exposure, toxicity, and other available information. Herein, we describe an approach that can be used to create a relevant PFAS landscape using the TSCA section 8(a)(7) rule definition to continue the efforts initiated in the National PFAS Testing Strategy.

A landscape of 13,054 PFAS substances was first created based on the structural definitions outlined in the TSCA rule. The landscape was then augmented with 2484 simulated degradation products of PFAS substances on the TSCA inventory using the Catalogic expert system. Adding simulated degradates was intended to enrich the landscape by substances that might be expected to be found in the environment from existing substances in commerce. The simulated degradation products were derived from an expert system which includes training set substances that are PFAS; however a full characterization of the model relative to the PFAS landscape was not feasible as some of the training set was proprietary in nature. For the portion of training set substances that could be evaluated – there was a minimal overlap in datasets as shown in Fig. S1. The robustness of the simulated degradation products is a limitation in the approach and requires additional work but a pragmatic one given the absence of data to refine and improve the model further.

The 15,525 substances in the PFAS landscape were then grouped into 128 terminal structural categories based on a stepwise process that combined OECD functional categories, chain length and structural similarity. The use of a chain length threshold of 7 was a pragmatic choice to help identify persistent long chain substances, though the subcategorization using this threshold may be best suited for straight chain linear PFAS. Some of the terminal categories were very large and structurally diverse whilst others showed much greater structural similarity highlighting both the complexity of the PFAS structural landscape and local areas of homogeneity.

A method was developed to select the most representative substance (centroid) and other substances (MaxMin) to capture the structural diversity of each terminal category and guide data collection efforts. A substantial number of representative and diverse substances (~6000) would be required to capture 80% percent of structural diversity in the terminal categories for the unconstrained landscape. Significantly fewer representative and diverse substances (101) would be required to capture 80% percent of structural diversity in the terminal categories for the TSCA constrained landscape (though if ToxVal data availability was factored in, this would reduce to 76 substances). The difference in utilizing the unconstrained and TSCA constrained landscape highlights the challenges in data collection to address future and theoretical data gaps versus those data gaps that exist amongst substances known to be in commerce.

Publicly available *in vivo* data across the terminal categories were used to evaluate whether read-across could be potentially viable based on the variation of the *in vivo* data itself. A 25^th^ percentile of the derived human equivalent BMDs served as surrogate POD value for a given substance. Not all terminal categories were associated with toxicity data but for those categories, the following insights were noted; substances with very short or long carbon chain length within a category tended to exhibit lower toxicities (i.e., higher PODs), but the spread of PODs within a category could be large particularly for diverse categories based on carbon chain length, spanning 1–2 orders of magnitude or more. In addition to the shift in potency between terminal categories containing longer vs shorter chain lengths, there was also a shift between terminal categories with different functional groups e.g. Aromatic PFASs tended to be less potent vs. PFAAs. The variability of *in vivo* PODs from traditional toxicity tests within some of the terminal categories suggests that structural considerations may not be sufficient for performing read across without additional data collection.

Information from NAMs were layered on the terminal categories to help identify potentially distinct mechanistic and TK-related subgroups. The NAM information was intended to help refine terminal categories, guide candidate selection, and inform data collection efforts. Currently, the terminal categories and candidates for data collection are primarily identified based on chemical structural considerations; however, other factors such as toxicokinetics, hazard, and modes-of-action are also important when considering a category and read-across approach [22]. To incorporate these other factors, the current process for selecting representative and diverse substances (i.e., centroid, MaxMin) could also be applied to the mechanistic and TK-related subgroups to select candidates for data collection as well as inform the types of tests that may be useful in characterizing the hazards associated with a specific terminal category. For example, subgroups predicted to have endocrine-related activity may benefit from developmental and reproductive tests, whilst those predicted to have significant cross-species TK differences may have limited benefits from rodent-based TK studies. While the current NAM data and enrichment flags are limited, the performance of these models will improve over time as additional substances are tested. Similar approaches have been proposed or incorporated into case studies, but not operationalized for large groups of chemicals [20,59,60]. Information from environmental measurements and release, traditional toxicity data, and chemical properties (physical state and physicochemical properties) were also layered on the terminal categories to help identify priorities for further data collection efforts.

The landscape of PFAS substances is substantially large and diverse with limited human health data. A category approach enables strategic data collection with the longer-term goal of enabling read-across within a particular category. In an effort to address this goal, a stepwise systematic process was developed to group the substances using a combination of OECD primary categories, sequential fluorinated carbon chain length, and structural similarity. The process attempted to balance maximizing structural similarity within each category relative to a manageable number of categories. Within each category, representative substances to capture the structural diversity were identified to guide data collection efforts. A substantial number of substances were required to capture a large percentage of structural diversity in the terminal categories for the full PFAS landscape, whilst a significantly fewer number were needed to capture the structural diversity for the TSCA active constrained landscape. The difference in utilizing the full and TSCA active constrained PFAS landscape highlights the challenges in data collection to address future and theoretical data gaps versus those data gaps that exist amongst substances known to be in commerce. To assist in prioritizing the categories for data collection, information from environmental measurements and release, traditional toxicity data, and exposure considerations based on chemical properties were incorporated to focus on those categories of greatest need. The variability in POD values from existing traditional *in vivo* toxicity tests within some of the terminal categories suggests that the structural considerations may not be sufficient for performing read-across without additional data collection. TK information is another important factor that can help resolve the variability observed. It should also be noted that POD values are one of many considerations – a reference dose effect will likely vary across similar chemicals within a category even if they exhibit similar hazard profiles. Information from NAMs were used to help refine the terminal categories based on potentially distinct mechanistic and TK-related subgroups and inform the types of data collection activities that may be required. Finally, a machine learning modelling approach was applied in an attempt to build a predictive model to operationalize the terminal categories developed, such that new PFAS not already captured in the landscape could be profiled and assigned to their most probable terminal category. The methods developed for categorizing the PFAS landscape, selecting representative substances, refining categories based on mechanistic and TK information, and prioritizing categories for data collection provide a robust foundation to aid EPA in addressing the significant challenges associated with evaluating the environmental and human health impacts of this class of chemicals. The methods and associated categories are flexible in accommodating additional data as it is generated and may evolve as the scientific knowledge grows.

## Supplementary Material

s1

SI2

SI2 data

SI2 pfas data

SI2 performance

## Figures and Tables

**Table 1:** List of PFAS-Atlas class assignments and the corresponding primary categories used in this study. Si PFAS refer to Silicon PFAS, HFCs refer to hydrofluorocarbons, PASF-based substances refer to perfluoroalkane sulfonyl fluorides, PFAA refer to perfluoroalkyl acids and PolyFACs are polyfluoroalkyl alcohols. Substances that could not be positively categorized by PFAS-Atlas were denoted as ‘other’.

PFAS-Atlas first class	Primary category

PFAAs	PFAAs
PFAAs, cyclic	PFAAs, cyclic
PFAA precursors	PFAA precursors
PFAA precursors	PASF-based substances
PFAA precursors	n:2 fluorotelomer-based substances
PFAA precursors	HFCs
PFAA precursors, cyclic	PFAA precursors, cyclic
Polyfluoroalkyl acids	Polyfluoroalkyl acids
Polyfluoroalkyl acids	PolyFCA derivatives
Polyfluoroalkyl acids, cyclic	Polyfluoroalkyl acids, cyclic
Other PFAS	Other PFAS
Other PFAS	Aromatic PFASs
	Si PFASs
	Polyfluoroalkanes
	others*
Other PFAS,cyclic	Other PFAS,cyclic
Other PFAS,cyclic	others, cyclic*
Not PFAS	Unclassified

**Table 2:** Summary of NAM Flag Rationales.

Endpoint	Low Concern (Blue)	Medium Confidence (Yellow)	High Confidence (Red)

Nuclear Receptors	No nuclear receptor activity	Activity against at least one of the receptors ER, PPARA, PPARG, PPARD, NFE2L2, PXR, RARG, RXRB at the level of one or more samples in one assay.	Activity in the yellow medium concern that is confirmed in at least one sample in 2 orthogonal assays
DNT	No activity or activity was only observed at the highest concentration related to cytotoxicity	Low number of hits which demonstrated selective bioactivity	Moderate to high bioactivity (as measured by hitcall) and demonstrated selective bioactivity (activity below cytotoxicity AC50 as measured by AUC) and median AC50 < 10 μM
Zebrafish	Development was normal in all larvae	Test results were equivocal or if less than 50 % of the larvae were affected	Positive activity (i.e., elicited death, non-hatching, or malformations in at least 50 % of the animals)
Thyroid	No activity greater than 50 % of the model inhibitors/binders	Activity greater than 50 % of the model inhibitors/binder, but the concentration necessary to result in this activity was 2 orders of magnitude higher than the model inhibitors/binders	AC50s that were within 2 orders of magnitude of the model inhibitors/binders
Immune	Selectivity scores less than 0.25 log10 μM	Selectivity scores of greater than 0.25 log10 μM	
TK Plasma Binding (TK_PlasBind)	TK_PlasBInd_High: Plasma protein binding higher than 50 % of non-PFAS chemicals (f_up < 0.11) (this corresponds to 25th percentile of PFAS (fup < 0.10)	TK_PlasBInd_Higher: Plasma protein binding higher than 50 % of PFAS chemicals (f_up < 0.0109)	TK_PlasBInd_Highest: Plasma protein binding higher than 75 % of PFAS (f_up < 0.0039)
TK Instrinic Clearance (TK_Metab)	TK_Metab_Moderate: Clint in upper 75th percentile of exp PFAS data (Clint > 5.97 ul/min/million cells). Max Clint = 49.86	TK_Metab_Slow: Clint < 5.97 ul/min/million heps. (lower 75th percentile)	TK_Metab_Stable: Stable in *in vitro* heptatocyte incubation (Clint = 0 or Clint pvalue > 0.05)
TK_Struc_Endo		Non-fluorinated structure is similar to endogenous chemicals. More likely to be a transporter substrate.	
TK half-life predictions	category 1 or 2 to denote half-life ≤ 12 h, or 12 h – 1 week	category 3 = 1 week – 2 months	category 4 ≥ 2 months

**Table 3:** List of secondary categories exceeding the threshold and their corresponding median pairwise distances (rounded to 2 decimal places).

Primary-Secondary Categories	Median Within-Category Pairwise distance

others, cyclic, lt7	0.92
PFAA precursors, cyclic, lt7	0.90
Other PFASs, cyclic, lt7	0.89
Aromatic PFASs, lt7	0.88
Other PFASs, lt7	0.88
unclassified, lt7	0.87
others, lt7	0.87
Polyfluoroalkyl acids, cyclic, lt7	0.86
PFAA precursors, lt7	0.86
Other PFASs, cyclic, gte7	0.85
Polyfluoroalkanes, lt7	0.85
PolyFCA derivatives, lt7	0.83
PFAAs, lt7	0.82
PFAAs, cyclic, lt7	0.82
Polyfluoroalkyl acids, lt7	0.81
HFCs, lt7	0.81

**Table 4:** Terminal categories for which the MaxMin approach was not undertaken.

Terminal category	Membership
Other PFASs, cyclic, gte7, 1	2
Other PFASs, cyclic, gte7, 2	4
Other PFASs, cyclic, gte7, 3	2
PFAAs, cyclic, gte7	1
PFAAs, cyclic, lt7, 3, 1	4
unclassified, lt7, 2.0, 1.0	5
unclassified’, lt7, 2.0, 2.0	2

**Table 5:** Terminal categories for which 3 representative substance selections capture more than 50% of the structural diversity.

Terminal category	Number of chemicals for 80 % structural diversity	Cumulative % of Structural Diversity	Terminal Category size

Other PFASs, cyclic, gte7, 4	3	84.03	6
Polyfluoroalkyl acids, cyclic, lt7, 3	3	83.67	11
unclassified, lt7, 1.0, 1.0	4	67.35	8
Other PFASs, cyclic, lt7, 1.0, 2.0	4	67.14	9
PFAAs, cyclic, lt7, 3.0, 2.0	5	64.87	9
others, cyclic, lt7, 2.0, 3.0	5	63.47	10
others, cyclic, lt7, 3.0, 4.0	5	62.24	13
PFAAs, cyclic, lt7, 2.0	6	59.02	14
others, cyclic, lt7, 2.0, 1.0	5	58.79	10
PFAA precursors, cyclic, lt7, 1.0, 1.0	6	58.03	19
PFAAs, cyclic, lt7, 3.0, 3.0	6	57.47	12
Polyfluoroalkyl acids, cyclic, lt7, 4.0	6	55.93	12
unclassified, lt7, 3.0, 2.0	6	55.75	11
others, cyclic, lt7, 1.0, 1.0	6	55.17	12
Polyfluoroalkyl acids, cyclic, lt7, 1.0	6	54.9	15

Notes: Column 1 represents the number of substances that would be required to capture 80% of the structural diversity in the category, Cumulative % of Structural Diversity represents the normalized cumulative minimum distance for up to 3 selected diverse substances. Note the 80% used as a threshold is purely for illustrative purposes only.

**Table 6:** Number (percentage) of substances assigned to each physical state and physicochemical designation.

Physical state and physicochemical designation	Full landscape	TSCA active constrained landscape

A (insoluble solids)	2060 (13.2 %)	25 (12.6 %)
B (soluble solids and soluble non-volatile liquids)	9824 (63.3 %)	71 (35.7 %)
C (soluble volatile liquids/insoluble liquids and soluble gases)	3115 (20 %)	85 (42.7 %)
D (insoluble gases or highly volatile gases)	95 (0.6 %)	10 (5 %)
No designation	431 (2.8 %)	8 (4 %)

**Table 7:** Performance metrics for enriched PFAS ToxPrints.

comparison	ROC_AUC_score	Sensitivity	Specificity

[NR_ESR1, pred_NR_ESR1]	0.67	0.47	0.87
[NR_PPAR, pred_NR_PPAR]	0.78	0.88	0.68
[NR_NRF2, pred_NR_NRF2]	0.82	1.00	0.64
[NR_PXR, pred_NR_PXR]	0.76	1.00	0.53
[ZF, pred_ZF]	0.66	0.59	0.74
[DNT, pred_DNT]	0.56	0.14	0.98
[Immune, pred_Immune]	0.55	0.10	0.99
[TK_PlasBind, pred_TK_PlasBind]	0.71	0.78	0.63

**Table 8:** Terminal categories from the constrained TSCA active landscape where the MaxMin approach had been applied. Terminal categories for which 3 representative substance selections capture more than 50% of the structural diversity.

Terminal category	Number of chemicals for 80 % structural diversity	Cumulative % of structural diversity	Terminal category size

n:2 fluorotelomer-based substances, lt7	4	79.39	7
Other PFASs, lt7, 3.0, 1.0	3	87.82	6
Other PFASs, cyclic, lt7, 2.0	4	75.73	8
PASF-based substances, gte7	6	50.43	18
PFAA precursors, gte7	4	66.79	19
PFAA precursors, lt7, 2.0, 2.0	3	83.96	6
PFAA precursors, lt7, 2.0, 3.0	2	97.06	6
PFAA precursors, lt7, 3.0, 2.0	5	66.84	9
PFAA precursors, lt7, 4.0, 2.0	5	63.06	11
PFAAs, gte7	3	90	11
PFAAs, lt7, 1.0	3	81.61	8
PFAAs, lt7, 4.0	5	59.97	12
n:2 fluorotelomer-based substances, gte7	1	100	6

Notes: ‘Number of chemicals for 80% structural diversity’ represents the number of diverse substances that would need to be selected to capture 80% of the structural diversity, ‘Cumulative % of Structural Diversity’ reflects the structural diversity captured by the up to 3 diverse substances already made and ‘Terminal Category size’ reflects the size of the terminal category if constrained by the availability of TSCA active substances.

## Footnotes

2 Note the approach taken in Aurisano is not necessarily consistent with uncertainty factor selection information provided in EPA’s 2002 Review of the Reference Dose and Reference Concentration Processes and EPA’s 2022 ORD Staff Handbook for Developing IRIS Assessments.

## References

[1] Wang Z, DeWitt JC, Higgins CP, Cousins IT, A never-ending story of per- and polyfluoroalkyl substances (PFASs)? Environ. Sci. Technol. 51 (2017) 2508–2518, 10.1021/acs.est.6b04806. 28224793

[2] Glüge J, Scheringer M, Cousins IT, DeWitt JC, Goldenman G, Herzke D, Lohmann R, Ng CA, Trier X, Wang Z, An overview of the uses of per- and polyfluoroalkyl substances (PFAS), Environ. Sci. Processes Impacts 22 (2020) 2345–2373, 10.1039/D0EM00291G. PMC7784712 33125022

[3] Gaines LGT, Historical and current usage of per- and polyfluoroalkyl substances (PFAS): a literature review, Am. J. Ind. Med. 66 (2023) 353–378, 10.1002/ajim.23362. 35614869

[4] Wallis DJ, Barton KE, Knappe DRU, Kotlarz N, McDonough CA, Higgins CP, Hoppin JA, Adgate JL, Source apportionment of serum PFASs in two highly exposed communities, Sci. Total Environ. 855 (2023) 158842, 10.1016/j.scitotenv.2022.158842. 36122706 PMC10564447

[5] Chen Y, Zhang H, Liu Y, Bowden JA, Tolaymat TM, Townsend TG, Solo-Gabriele HM, Evaluation of per- and polyfluoroalkyl substances (PFAS) in leachate, gas condensate, stormwater and groundwater at landfills, Chemosphere 318 (2023) 137903, 10.1016/j.chemosphere.2023.137903. 36669537 PMC10536789

[6] Li J, Xi B, Zhu G, Yuan Y, Liu W, Gong Y, Tan W, A critical review of the occurrence, fate and treatment of per- and polyfluoroalkyl substances (PFASs) in landfills, Environ. Res. 218 (2023) 114980, 10.1016/j.envres.2022.114980. 36460077

[7] Bolan N, Sarkar B, Vithanage M, Singh G, Tsang DCW, Mukhopadhyay R, Ramadass K, Vinu A, Sun Y, Ramanayaka S, Hoang SA, Yan Y, Li Y, Rinklebe J, Li H, Kirkham MB, Distribution, behaviour, bioavailability and remediation of poly- and per-fluoroalkyl substances (PFAS) in solid biowastes and biowaste-treated soil, Environ. Int. 155 (2021) 106600, 10.1016/j.envint.2021.106600. 33964642

[8] OECD, Reconciling Terminology of the Universe of Per- and Polyfluoroalkyl Substances: Recommendations and Practical Guidance, Series on Risk Management No. 61., 2021.

[9] Gaber N, Bero L, Woodruff TJ, The devil they knew: chemical documents analysis of industry influence on PFAS science, Ann. Global Health, 89 (>n.d.) 37. 10.5334/aogh.4013. PMC10237242 37273487

[10] Wang Z, Buser AM, Cousins IT, Demattio S, Drost W, Johansson O, Ohno K, Patlewicz G, Richard AM, Walker GW, White GS, Leinala E, A new OECD definition for per- and polyfluoroalkyl substances, Environ. Sci. Tech. 55 (2021) 15575–15578, 10.1021/acs.est.1c06896. 34751569

[11] Grulke CM, Williams AJ, Thillanadarajah I, Richard AM, EPA’s DSSTox database: history of development of a curated chemistry resource supporting computational toxicology research, Computat. Toxicol. (Amsterdam, Netherlands) 12 (2019), 10.1016/j.comtox.2019.100096. PMC7787967 33426407

[12] Schymanski EL, Zhang J, Thiessen PA, Chirsir P, Kondic T, Bolton EE, Per- and Polyfluoroalkyl Substances (PFAS) in PubChem: 7 Million and Growing, Environ. Sci. Tech. (2023), 10.1021/acs.est.3c04855. PMC10634333 37871188

[13] US EPA, Per- and Poly-Fluoroalkyl Chemical Substances Designated as Inactive on the TSCA Inventory, Significant New Use Rule, Federal Register, 2023 https://www.federalregister.gov/documents/2023/01/26/2023-01156/per–and-poly-fluoroalkyl-chemical-substances-designated-as-inactive-on-the-tsca-inventory (accessed September 11, 2023.

[14] US EPA, Federal Register (2023). https://www.federalregister.gov/documents/2023/10/11/2023-22094/toxic-substances-control-act-reporting-and-recordkeeping-requirements-for-perfluoroalkyl-and.

[15] US EPA, Federal Register (2024). https://www.federalregister.gov/documents/2024/01/11/2024-00412/per–and-poly-fluoroalkyl-chemical-substances-designated-as-inactive-on-the-tsca-inventory.

[16] Carlson LM, Angrish M, Shirke AV, Radke EG, Schulz B, Kraft A, Judson R, Patlewicz G, Blain R, Lin C, Vetter N, Lemeris C, Hartman P, Hubbard H, Arzuaga X, Davis A, Dishaw LV, Druwe IL, Hollinger H, Jones R, Kaiser JP, Lizarraga L, Noyes PD, Taylor M, Shapiro AJ, Williams AJ, Thayer KA, Systematic Evidence Map for Over One Hundred and Fifty Per- and Polyfluoroalkyl Substances (PFAS), Environ. Health Perspect. 130 (2022) 056001, 10.1289/EHP10343. 35580034 PMC9113544

[17] U. EPA, Status and future directions of the high production volume challenge program, (2004). https://nepis.epa.gov/Exe/ZyNET.exe/P1004QXK.TXT?ZyActionD=ZyDocument&Client=EPA&Index=2000+Thru+2005&Docs=&Query=&Time=&EndTime=&SearchMethod=1&TocRestrict=n&Toc=&TocEntry=&QField=&QFieldYear=&QFieldMonth=&QFieldDay=&IntQFieldOp=0&ExtQFieldOp=0&XmlQuery=&File=D%3A%5Czyfiles%5CIndex%20Data%5C00thru05%5CTxt%5C00000021%5CP1004QXK.txt&User=ANONYMOUS&Password=anonymous&SortMethod=h%7C-&MaximumDocuments=1&FuzzyDegree=0&ImageQuality=r75g8/r75g8/x150y150g16/i425&Display=hpfr&DefSeekPage=x&SearchBack=ZyActionL&Back=ZyActionS&BackDesc=Results%20page&MaximumPages=1&ZyEntry=1&SeekPage=x&ZyPURL# (accessed October 12, 2023).

[18] OECD, Guidance on Grouping of Chemicals, Second Edition OECD Series on Testing and Assessment, No 194, OECD Publishing (2017). 10.1787/9789264274679-en.

[19] Cronin MTD, Chapter 1:An Introduction to Chemical Grouping, Categories and Read-Across to Predict Toxicity, in: Chemical Toxicity Prediction, 2013: pp. 1–29. 10.1039/9781849734400-00001.

[20] Escher SE, Kamp H, Bennekou SH, Bitsch A, Fisher C, Graepel R, Hengstler JG, Herzler M, Knight D, Leist M, Norinder U, Ouédraogo G, Pastor M, Stuard S, White A, Zdrazil B, van de Water B, Kroese D, Towards grouping concepts based on new approach methodologies in chemical hazard assessment: The read-across approach of the EU-ToxRisk project, Arch. Toxicol. 93 (2019) 3643–3667, 10.1007/s00204-019-02591-7. 31781791

[21] Patlewicz G, Cronin MTD, Helman G, Lambert JC, Lizarraga LE, Shah I, Navigating through the minefield of read-across frameworks: a commentary perspective, Comput. Toxicol. 6 (2018) 39–54, 10.1016/j.comtox.2018.04.002.

[22] Patlewicz G, Shah I, Towards systematic read-across using Generalised Read-Across (GenRA), Comput. Toxicol. 25 (2023) 100258, 10.1016/j.comtox.2022.100258. PMC10483627 37693774

[23] Patlewicz G, Richard AM, Williams AJ, Judson RS, Thomas RS, Towards reproducible structure-based chemical categories for PFAS to inform and evaluate toxicity and toxicokinetic testing, Comput. Toxicol. 24 (2022) 100250, 10.1016/j.comtox.2022.100250. PMC10031514 36969381

[24] Stucki AO, Barton-Maclaren TS, Bhuller Y, Henriquez JE, Henry TR, Hirn C, Miller-Holt J, Nagy EG, Perron MM, Ratzlaff DE, Stedeford TJ, Clippinger AJ, Use of new approach methodologies (NAMs) to meet regulatory requirements for the assessment of industrial chemicals and pesticides for effects on human health, Front. Toxicol. 4 (2022), 10.3389/ftox.2022.964553. PMC9475191 36119357

[25] Carstens KE, Freudenrich T, Wallace K, Choo S, Carpenter A, Smeltz M, Clifton MS, Henderson WM, Richard AM, Patlewicz G, Wetmore BA, Paul Friedman K, Shafer T, Evaluation of Per- and Polyfluoroalkyl Substances (PFAS) In Vitro Toxicity Testing for Developmental Neurotoxicity, Chem. Res. Toxicol. 36 (2023) 402–419, 10.1021/acs.chemrestox.2c00344. 36821828 PMC10249374

[26] Houck KA, Patlewicz G, Richard AM, Williams AJ, Shobair MA, Smeltz M, Clifton MS, Wetmore B, Medvedev A, Makarov S, Bioactivity profiling of per- and polyfluoroalkyl substances (PFAS) identifies potential toxicity pathways related to molecular structure, Toxicology 457 (2021) 152789, 10.1016/j.tox.2021.152789. 33887376

[27] Houck KA, Friedman KP, Feshuk M, Patlewicz G, Smeltz M, Clifton MS, Wetmore BA, Velichko S, Berenyi A, Berg EL, Evaluation of 147 perfluoroalkyl substances for immunotoxic and other (patho)physiological activities through phenotypic screening of human primary cells, ALTEX 40 (2023) 248–270, 10.14573/altex.2203041. 36129398 PMC10331698

[28] Kreutz A, Clifton MS, Henderson WM, Smeltz MG, Phillips M, Wambaugh JF, Wetmore BA, Category-Based Toxicokinetic Evaluations of Data-Poor Per- and Polyfluoroalkyl Substances (PFAS) using Gas Chromatography Coupled with Mass Spectrometry, Toxics 11 (2023) 463, 10.3390/toxics11050463. 37235277 PMC10223284

[29] Smeltz M, Wambaugh JF, Wetmore BA, Plasma Protein Binding Evaluations of Per- and Polyfluoroalkyl Substances for Category-Based Toxicokinetic Assessment, Chem. Res. Toxicol. (2023), 10.1021/acs.chemrestox.3c00003. PMC10506455 37184865

[30] Smeltz MG, Clifton MS, Henderson WM, McMillan L, Wetmore BA, Targeted Per- and Polyfluoroalkyl substances (PFAS) assessments for high throughput screening: Analytical and testing considerations to inform a PFAS stock quality evaluation framework, Toxicol. Appl. Pharmacol. 459 (2023) 116355, 10.1016/j.taap.2022.116355. 36535553 PMC10367912

[31] Stoker TE, Wang J, Murr AS, Bailey JR, Buckalew AR, High-Throughput Screening of ToxCast PFAS Chemical Library for Potential Inhibitors of the Human Sodium Iodide Symporter, Chem. Res. Toxicol. 36 (2023) 380–389, 10.1021/acs.chemrestox.2c00339. 36821091 PMC12050117

[32] Degitz SJ, Olker JH, Denny JS, Degoey PP, Hartig PC, Cardon MC, Eytcheson SA, Haselman JT, Mayasich SA, Hornung MW, In vitro screening of per- and polyfluorinated substances (PFAS) for interference with seven thyroid hormone system targets across nine assays, Toxicology in Vitro 95 (2024) 105762, 10.1016/j.tiv.2023.105762. 38072180 PMC11081714

[33] Williams AJ, Grulke CM, Edwards J, McEachran AD, Mansouri K, Baker NC, Patlewicz G, Shah I, Wambaugh JF, Judson RS, Richard AM, The CompTox Chemistry Dashboard: A community data resource for environmental chemistry, J. Cheminf. 9 (2017) 61, 10.1186/s13321-017-0247-6. PMC5705535 29185060

[34] Heller SR, McNaught A, Pletnev I, Stein S, Tchekhovskoi D, InChI, the IUPAC International Chemical Identifier, J. Cheminf. 7 (2015) 23, 10.1186/s13321-015-0068-4. PMC4486400 26136848

[35] Rogers D, Hahn M, Extended-Connectivity Fingerprints, J. Chem. Inf. Model. 50 (2010) 742–754, 10.1021/ci100050t. 20426451

[36] McInnes L, Healy J, Melville J, UMAP: Uniform manifold approximation and projection for dimension reduction, (2020). https://arxiv.org/abs/1802.03426.

[37] Dimitrov S, Dimitrova G, Pavlov T, Dimitrova N, Patlewicz G, Niemela J, Mekenyan O, A stepwise approach for defining the applicability domain of SAR and QSAR models, J. Chem. Inf. Model 45 (2005) 839–849, 10.1021/ci0500381. 16045276

[38] An Su., Cheng Y, Zhang C, Yang Y-F, She Y-B, Rajan Krishna, An artificial intelligence platform for automated PFAS subgroup classification: a discovery tool for PFAS screening, Sci. Total Environ. 921 (2024) 171229, 10.1016/j.scitotenv.2024.171229. 38402985

[39] Su A, Rajan K, A database framework for rapid screening of structure-function relationships in PFAS chemistry, Sci. Data 8 (2021) 14, 10.1038/s41597-021-00798-x. 33462239 PMC7814031

[40] Mansouri K, Grulke CM, Judson RS, Williams AJ, OPERA models for predicting physicochemical properties and environmental fate endpoints, J. Cheminf. 10 (2018) 10, 10.1186/s13321-018-0263-1. PMC5843579 29520515

[41] Chambers WS, Hopkins JG, Richards SM, A Review of per- and polyfluorinated alkyl substance impairment of reproduction, accessed July 5, 2023, Front. Toxicol. 3 (2021), https://www.frontiersin.org/articles/10.3389/ftox.2021.732436. 10.3389/ftox.2021.732436 PMC8915888 35295153

[42] Sznajder-Katarzyńska K, Surma M, Cieślik I, A Review of perfluoroalkyl acids (PFAAs) in terms of sources, applications, human exposure, dietary intake, toxicity, legal regulation, and methods of determination, J. Chem. 2019 (2019) e2717528.

[43] Richard AM, Hidle H, Patlewicz G, Williams AJ, Identification of branched and linear forms of PFOA and potential precursors: a user-friendly SMILES structure-based approach, accessed April 15, 2022, Front. Environ. Sci. 10 (2022), https://www.frontiersin.org/article/10.3389/fenvs.2022.865488. 10.3389/fenvs.2022.865488 PMC9048161 35494535

[44] Richard AM, Lougee R, Adams M, Hidle H, Yang C, Rathman J, Magdziarz T, Bienfait B, Williams AJ, Patlewicz G, A new CSRML structure-based fingerprint method for profiling and categorizing per- and polyfluoroalkyl substances (PFAS), Chem. Res. Toxicol. 36 (2023) 508–534, 10.1021/acs.chemrestox.2c00403. 36862450 PMC10031568

[45] Yang C, Tarkhov A, Marusczyk J, Bienfait B, Gasteiger J, Kleinoeder T, Magdziarz T, Sacher O, Schwab CH, Schwoebel J, Terfloth L, Arvidson K, Richard A, Worth A, Rathman J, New publicly available chemical query language, CSRML, to support chemotype representations for application to data mining and modeling, J. Chem. Inf. Model. 55 (2015) 510–528, 10.1021/ci500667v. 25647539

[46] Landrum GL, RDKit: Open-source cheminformatics, (n.d.). http://www.rdkit.org.

[47] O’Boyle NM, Sayle RA, Comparing structural fingerprints using a literature-based similarity benchmark, J. Cheminf. 8 (2016) 36, 10.1186/s13321-016-0148-0. PMC4932683 27382417

[48] Raymond JW, Blankley JC, Willett P, Comparison of chemical clustering methods using graph- and fingerprint-based similarity measures, J. Mol. Graphs Model. 21 (2003) 421–433, 10.1016/S1093-3263(02)00188-2. 12543138

[49] Ward JH, Hierarchical grouping to optimize an objective function, J. Am. Stat. Assoc. 58 (1963) 236–244, 10.1080/01621459.1963.10500845.

[50] Ashton M, Barnard J, Casset F, Charlton M, Downs G, Gorse D, Holliday J, Lahana R, Willett P, Identification of diverse database subsets using property-based and fragment-based molecular descriptions, Quant. Struct.-Act. Relat. 21 (2002) 598–604, 10.1002/qsar.200290002.

[51] Snarey M, Terrett NK, Willett P, Wilton DJ, Comparison of algorithms for dissimilarity-based compound selection, J. Mol. Graph. Model. 15 (1997) 372–385, 10.1016/s1093-3263(98)00008-4. 9704300

[52] Baker N, Knudsen T, Williams A, Abstract Sifter: A comprehensive front-end system to PubMed, F1000Research 6 (2017) Chem Inf Sci–2164. 10.12688/f1000research.12865.1. PMC5801564 29479422

[53] Aurisano N, Jolliet O, Chiu WA, Judson R, Jang S, Unnikrishnan A, Kosnik MB, Fantke P, Probabilistic points of departure and reference doses for characterizing human noncancer and developmental/reproductive effects for 10,145 chemicals, Environ. Health Perspect. 131 (2023) 037016, 10.1289/EHP11524. 36989077 PMC10056221

[54] Liberatore HK, Jackson SR, Strynar MJ, McCord JP, Solvent suitability for HFPO-DA (“GenX” Parent Acid) in toxicological studies, Environ. Sci. Technol. Lett. 7 (2020) 477–481, 10.1021/acs.estlett.0c00323. 32944590 PMC7490830

[55] Zhang C, McElroy AC, Liberatore HK, Alexander NLM, Knappe DRU, Stability of per- and polyfluoroalkyl substances in solvents relevant to environmental and toxicological analysis, Environ. Sci. Tech. 56 (2022) 6103–6112, 10.1021/acs.est.1c03979. PMC9065217 34734715

[56] Dawson DE, Lau C, Pradeep P, Sayre RR, Judson RS, Tornero-Velez R, Wambaugh JF, A machine learning model to estimate toxicokinetic half-lives of per- and polyfluoro-alkyl substances (PFAS) in multiple species, Toxics 11 (2023), 10.3390/toxics11020098. PMC9962572 36850973

[57] Wang J, Hallinger DR, Murr AS, Buckalew AR, Lougee RR, Richard AM, Laws SC, Stoker TE, High-throughput screening and chemotype-enrichment analysis of ToxCast phase II chemicals evaluated for human sodium-iodide symporter (NIS) inhibition, Environ. Int. 126 (2019) 377–386, 10.1016/j.envint.2019.02.024. 30826616 PMC9082575

[58] Pedregosa F, Varoquaux G, Gramfort A, Michel V, Thirion B, Grisel O, Blondel M, Prettenhofer P, Weiss R, Dubourg V, Vanderplas J, Passos A, Cournapeau D, Brucher M, Perrot M, Duchesnay É, Scikit-learn: machine learning in python, J. Mach. Learn. Res. 12 (2011) 28252830.

[59] Webster F, Gagné M, Patlewicz G, Pradeep P, Trefiak N, Judson R, Barton-Maclaren TS, Predicting estrogen receptor activation by a group of substituted phenols: an integrated approach to testing and assessment case study, Regul. Toxicol. Pharmacol. 106 (2019) 278–291, 10.1016/j.yrtph.2019.05.017. 31121201 PMC6786769

[60] OECD, Case study on the use of integrated approaches for testing and assessment IATA for estrogenicity of the substituted phenols, OECD Publishing, 2018.

