# Supplementary material for: Development of chemical categories for per- and polyfluoroalkyl substances (PFAS) and the proof-of-concept approach to the identification of potential candidates for tiered toxicological testing and human health assessment
Source: Comput Toxicol. Author manuscript; Available in PMC 2025 Sep 1. (PMC12181936; doi:10.1016/j.comtox.2024.100327)
Supplement: s1 [file NIHMS2067664-supplement-s1.docx]

Supplementary Information: Development of Chemical Categories for Per- and Polyfluoroalkyl Substances (PFAS) and the Proof-of-Concept Approach to the Identification of Potential Candidates for Tiered Toxicological Testing and Human Health Assessment

G. Patlewicz^1*^, R. S. Judson^1^, A. J. Williams^1^, T. Butler^2^, S. Barone Jr.^2^, K. E. Carstens^1^, J. Cowden^1^, J. L. Dawson^2^, S. J. Degitz^1^, K. Fay^2^, T. R. Henry^2^^, A. Lowit^2^, S. Padilla^1^, K. Paul Friedman^1^, M. B. Phillips^2^, D. Turk^2^, J. F. Wambaugh^1^, B. A Wetmore^1^, R. S. Thomas^1^

^1^Center for Computational Toxicology & Exposure (CCTE), U.S. Environmental Protection Agency, Research Triangle Park, Durham, NC, 27709, USA

^2^Office of Chemical Safety and Pollution Prevention (OSCPP), US Environmental Protection Agency, DC, USA

*Corresponding author. Grace Patlewicz Tel: +1 (919) 541-1540 Email: [patlewicz.grace@epa.gov](mailto:patlewicz.grace@epa.gov)

^Retired

# Supplementary information

## S.1 Evaluating the feasibility of subdividing the primary categories

Corina Symphony on the command line (licensed from Molecular Networks GmBH and Altamira LLC) was used to compute the 129 PFAS ToxPrints [44]. The Fisher’s exact test was used to compute an odds ratio and associated p value for each PFAS ToxPrint relative to the OECD primary category designation. This was comparable with the methodology discussed in Wang et al. [57]. A PFAS ToxPrint was considered enriched if it had an odds ratio greater than or equal to 3, a one-sided Fishers exact p-value less than 0.05 (probability value of the odds ratio being greater than 1) and the number of True Positives (TP) was determined to be greater than or equal to 3. For the “unclassified” primary category, the top enriched ToxPrints were PFAS bond and chain features including pfas_bond:S(=O)O_sulfonicAcid_acyclic_(chain)_SCF, pfas_chain:FT_n1_OP, pfas_chain:FT_n2_OP the latter represent fluorotelomer chains with either 1 or 2 CH2 units and an organophosphorus terminus. On the otherhand, the “PFAA precursors” had alcohols and carbonyls as enriched functional groups (pfas_bond:COH_alcohol_pri-alkyl_CF, pfas_bond:CC(=O)C_ketone_generic_CF). The intention was to explore whether certain types of features were specifically enriched in these broad primary categories to consider subcategorizing them to reduce the starting membership. The full set of enrichments for all primary categories are provided as a separate data file.

## Supplementary Figures

| 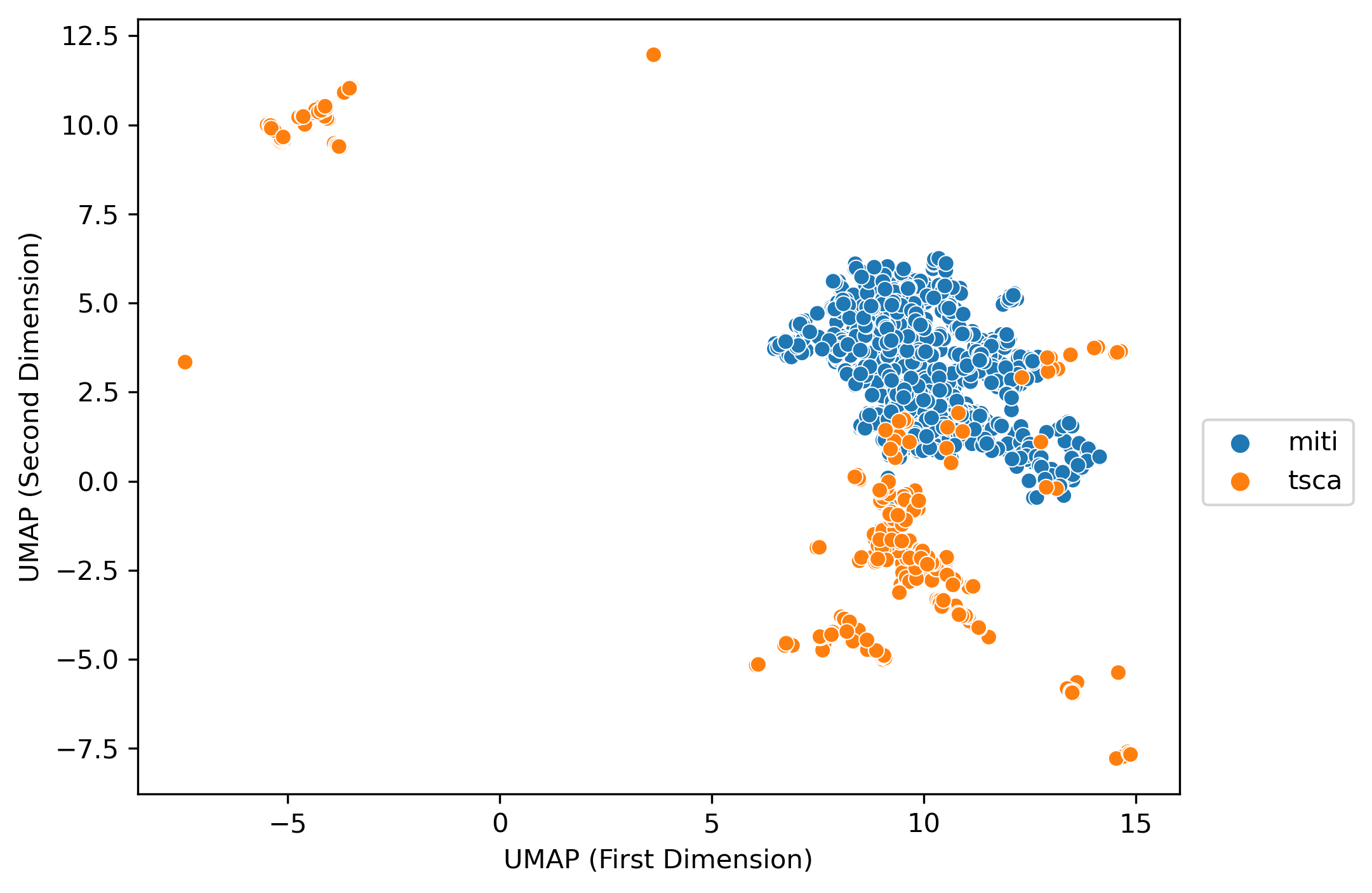  Figure S1: Overlap of MITI training data substances with TSCA substances using Morgan chemical fingers and represented in a UMAP plot |
| --- |

| 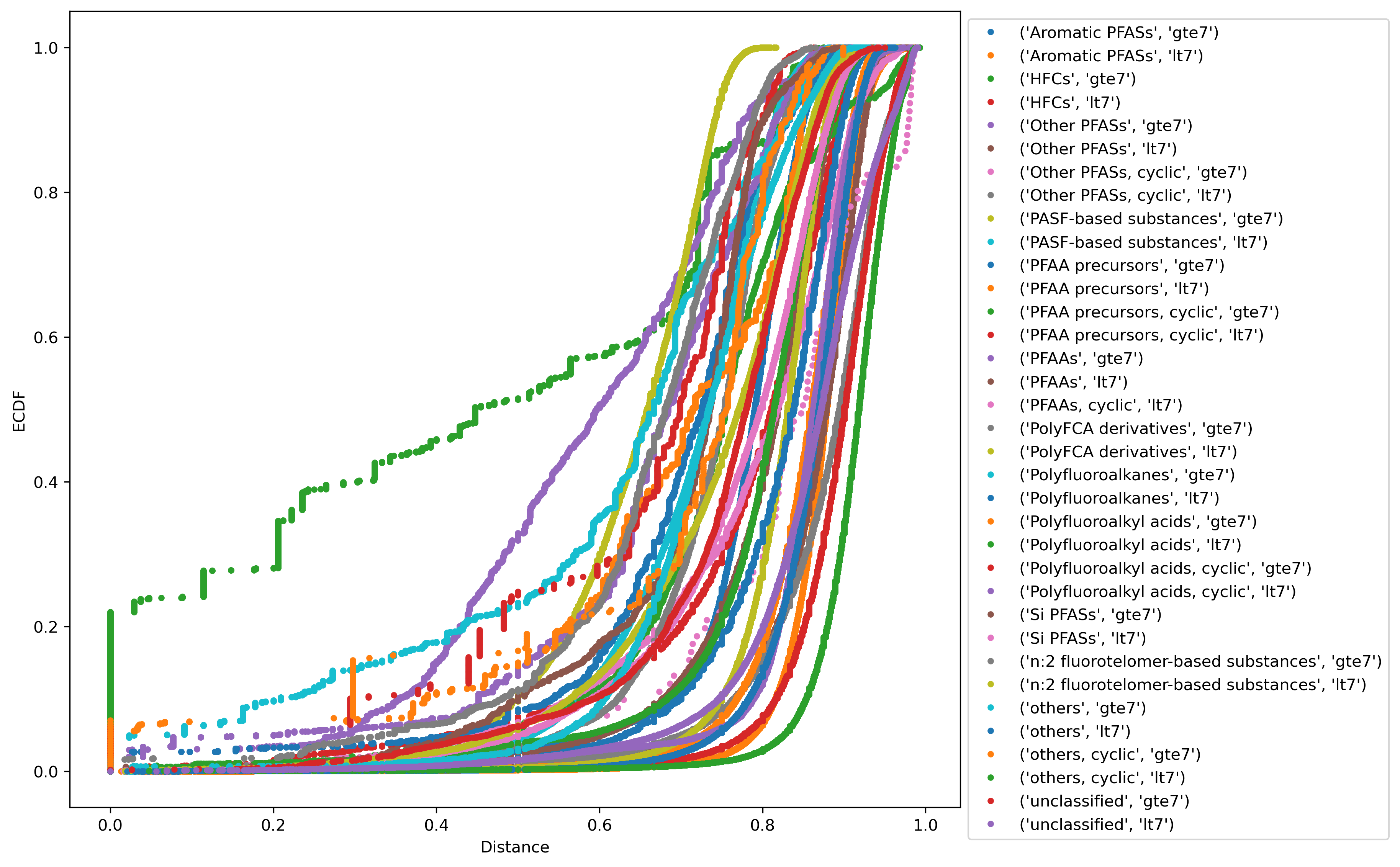  Figure S2: ECDFs of the within categories based on the chain length threshold of 7 |
| --- |

| 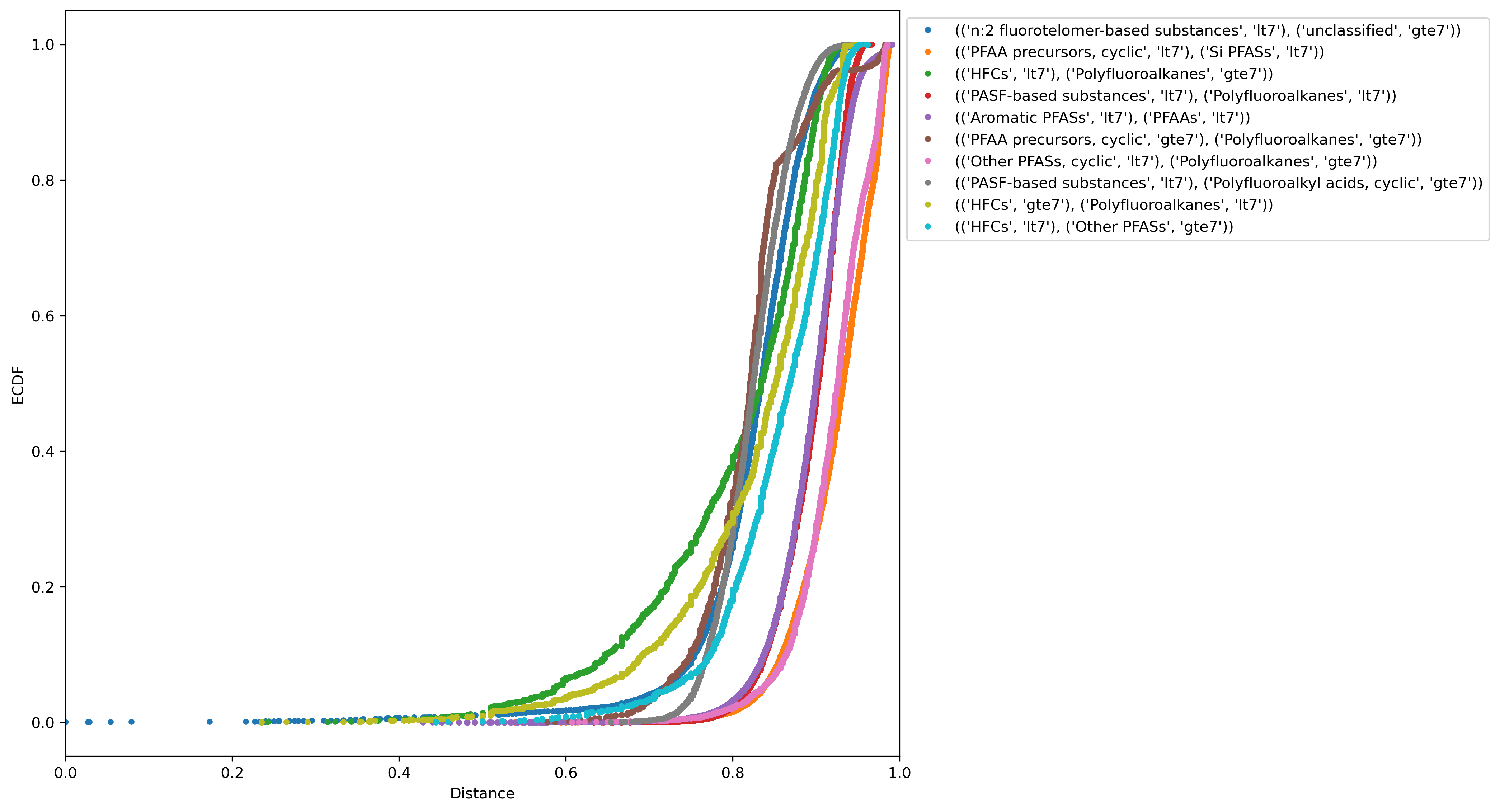  Figure S3: EDCFs for selected between category combinations for carbon chain length categories |
| --- |

| 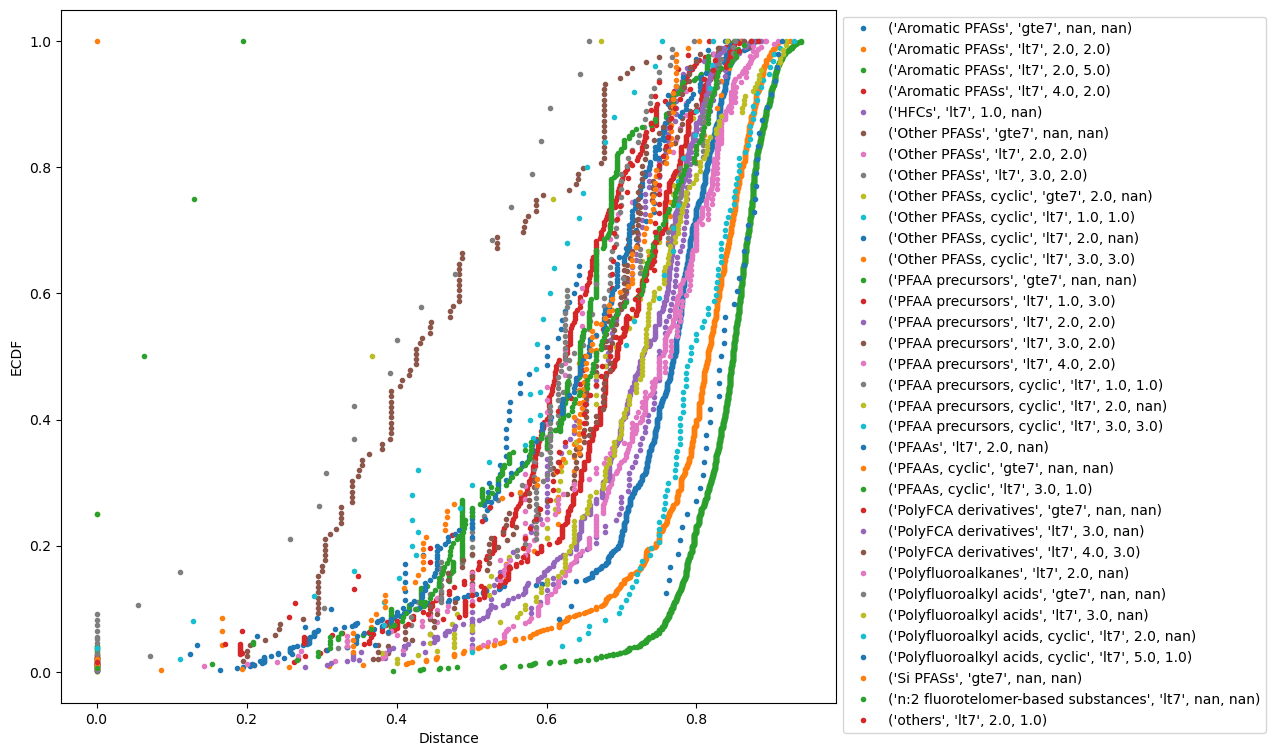  Figure S4: EDCFs for selected terminal categories to demonstrate left shift in pairwise distance |
| --- |

| 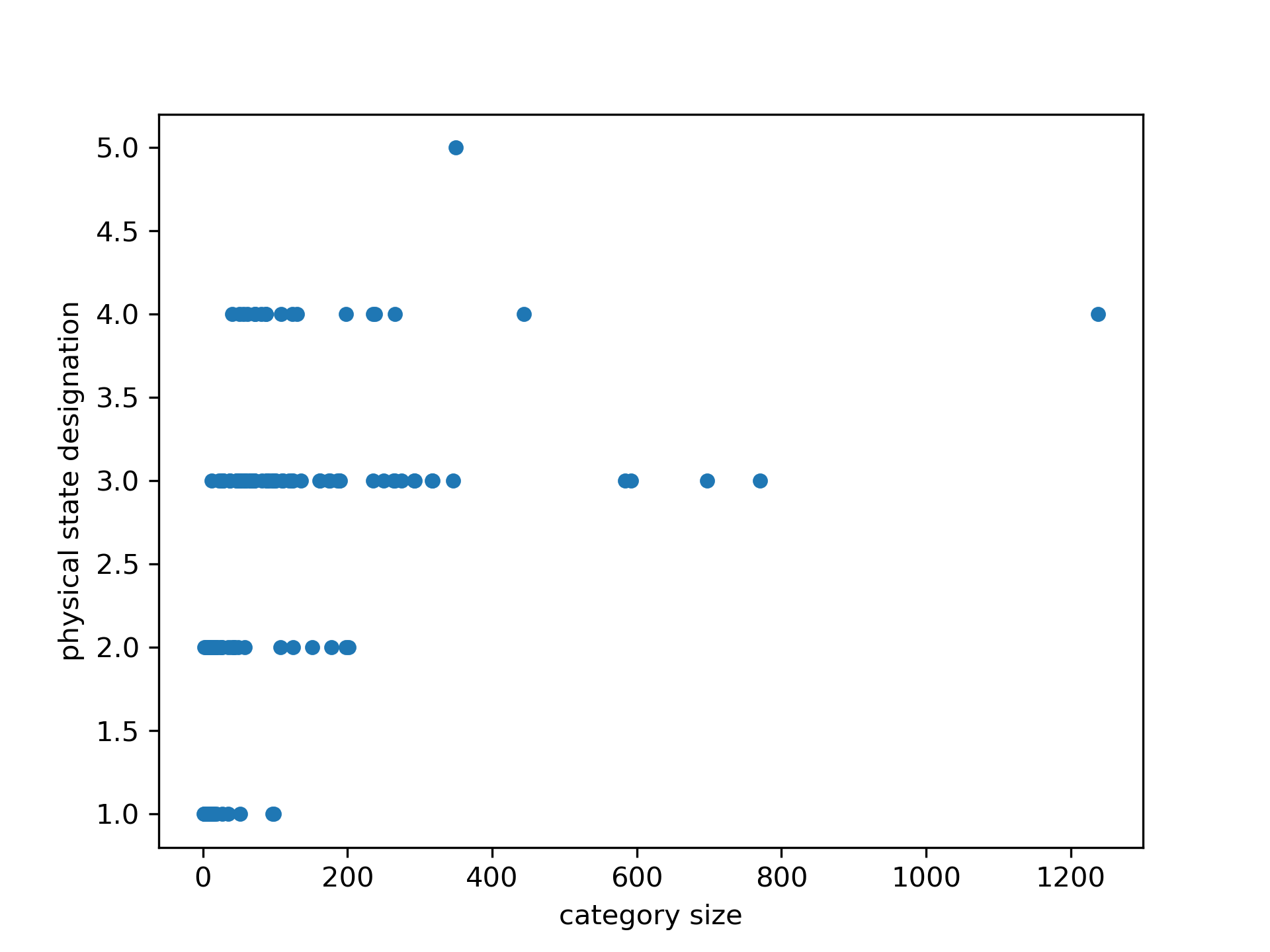  Figure S5: Correlation between terminal categories with large membership size and the number of designations represented amongst their memberships |
| --- |

| 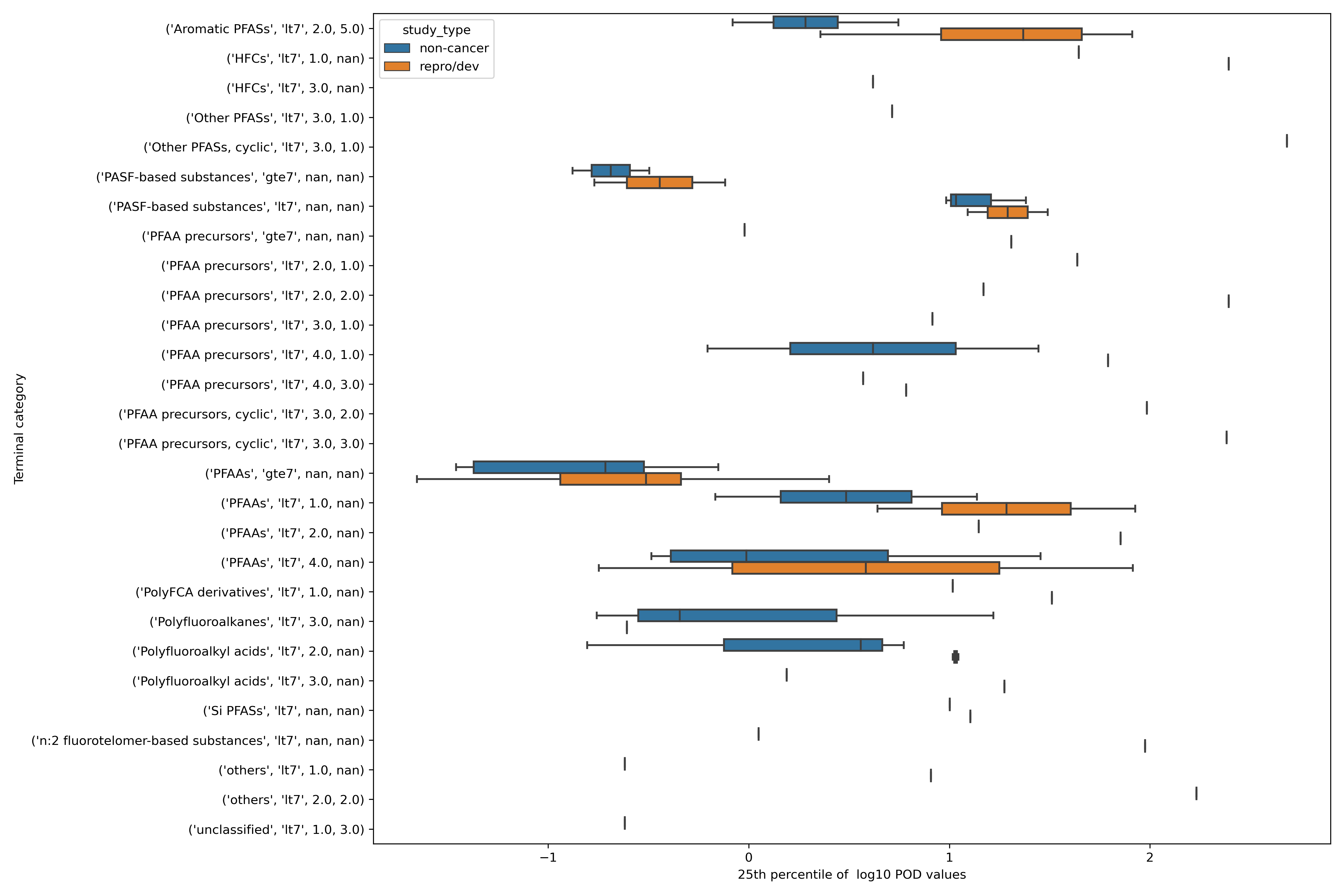  Figure S6: Boxplots of the variation of 25th percentiles of point of departure values (PODs) from non-cancer and repro/developmental studies. The box in the boxplot reflects the quartiles of the dataset, whilst the whiskers extend to + 1.5 * inter-quartile range (IQR). Outliers are shown as points if they exceed 1.5 * IQR. The repro/developmental boxplot is shown below the non-cancer boxplot for a given terminal category. |
| --- |

| 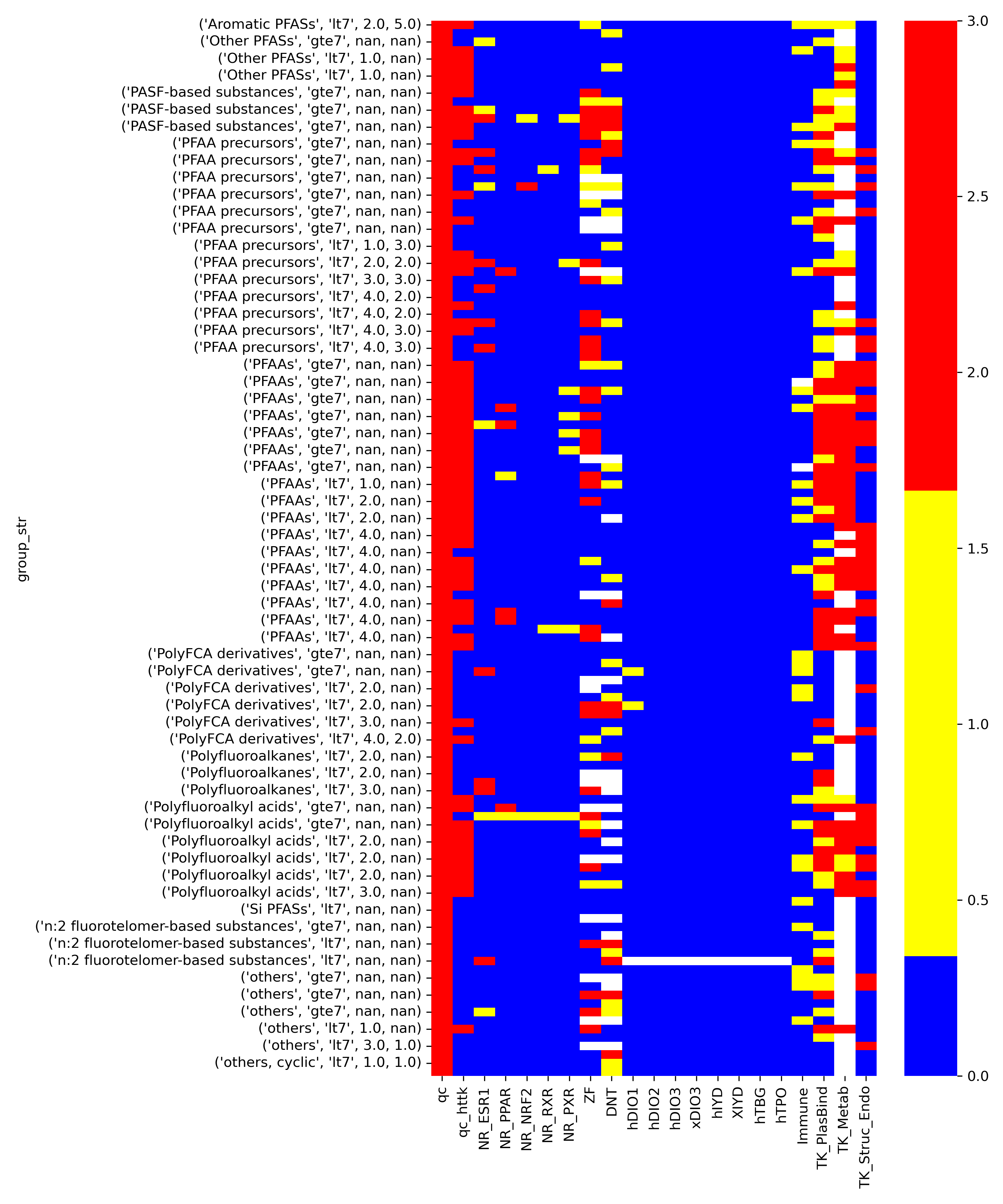  Figure S7: Heatmap of NAM flags for substances tested that overlap with the PFAS inventory |
| --- |


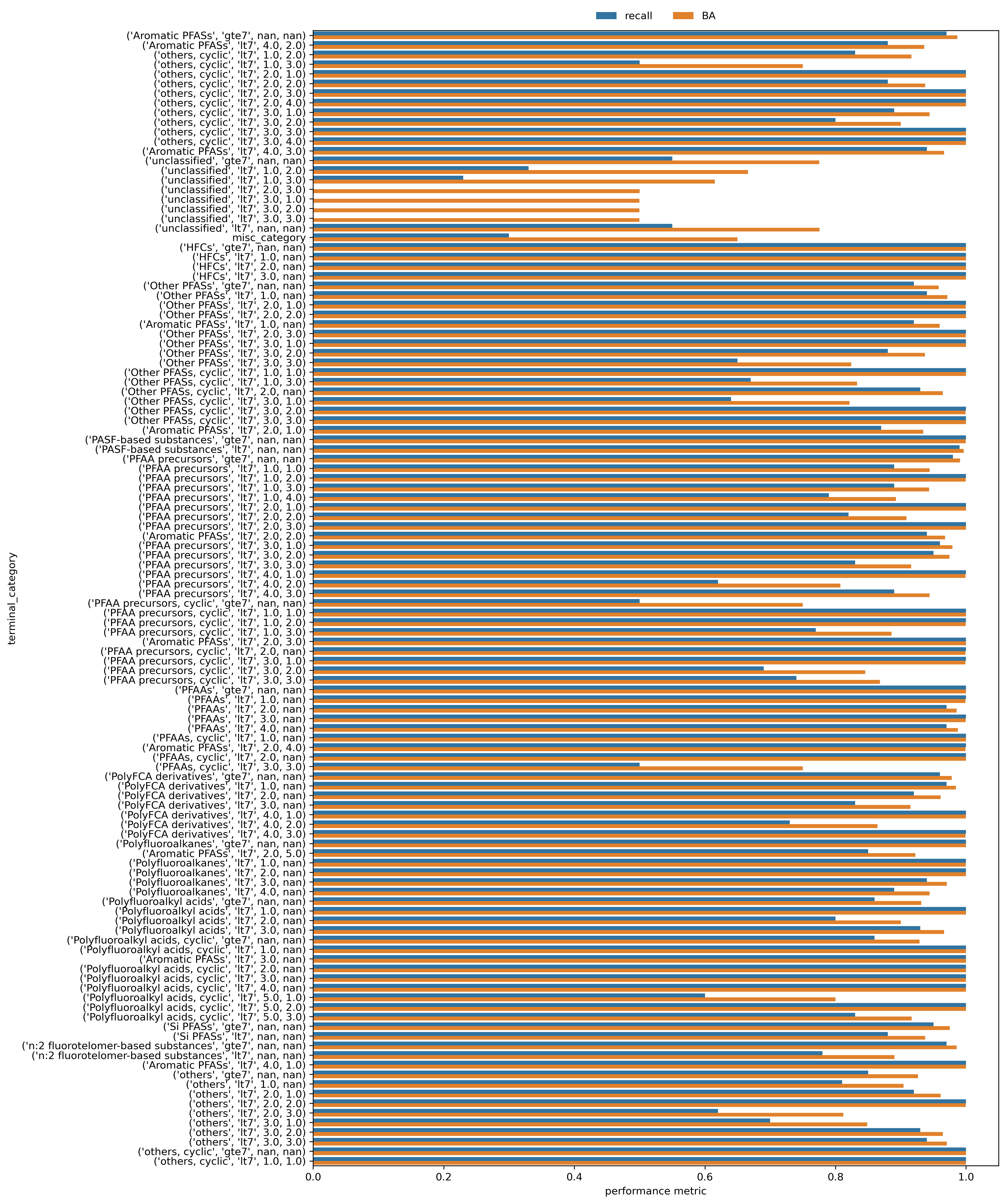


Figure S8: Barplot of the performance scores for the random forest classification model as applied to the hold out set of terminal categories
